# Bronze age Northern Eurasian genetics in the context of development of metallurgy and Siberian ancestry

**DOI:** 10.1038/s42003-024-06343-x

**Published:** 2024-06-11

**Authors:** Ainash Childebayeva, Fabian Fricke, Adam Benjamin Rohrlach, Lei Huang, Stephan Schiffels, Outi Vesakoski, Kristiina Mannermaa, Lena Semerau, Franziska Aron, Konstantin Solodovnikov, Marina Rykun, Vyacheslav Moiseyev, Valery Khartanovich, Igor Kovtun, Johannes Krause, Sergey Kuzminykh, Wolfgang Haak

**Affiliations:** 1 Department of Archaeogenetics, Max Planck Institute for Evolutionary Anthropology, D-04103 Leipzig, Germany; 2 Department of Anthropology, University of Texas at Austin, Austin, TX 78712 USA; 3 Department of Anthropology, University of Kansas, Lawrence, KS 66044 USA; 4 German Archaeological Institute, Eurasia Department, Berlin, 14195 Germany; 5 School of Computer and Mathematical Sciences, University of Adelaide, Adelaide, SA 5005 Australia; 6 Department of Finnish and Finno-Ugric Languages, University of Turku, Turku, 20014 Finland; 7 Department of Cultures, University of Helsinki, Yliopistonkatu 4, 00100 Helsinki, Finland; 8 Department of Archaeogenetics, Max Planck Institute for the Science of Human History, Jena, 07745 Germany; 9 Institute of Problems of Northern Development, Tyumen Scientific Center of the Siberian Branch of Russian Academy of Sciences, Tyumen, 625008 Russia; 10 Department of Anthropology and Ethnology, National Research Tomsk State University, Tomsk, 634050 Russia; 11 Peter the Great Museum of Anthropology and Ethnography (Kunstkamera), Russian Academy of Sciences, University Embankment, 3, Saint Petersburg, 199034 Russia; 12 Igor V. Kovtun, Independent Researcher, Kemerovo, 650000 Russia; 13 Russian Academy of Sciences, Institute of Archaeology, Laboratory of Natural Scientific Methods, Moscow, 117292 Russia

**Keywords:** Population genetics, Population genetics

## Abstract

The Eurasian Bronze Age (BA) has been described as a period of substantial human migrations, the emergence of pastoralism, horse domestication, and development of metallurgy. This study focuses on two north Eurasian sites sharing Siberian genetic ancestry. One of the sites, Rostovka, is associated with the Seima-Turbino (ST) phenomenon (~2200-1900 BCE) that is characterized by elaborate metallurgical objects found throughout Northern Eurasia. The genetic profiles of Rostovka individuals vary widely along the forest-tundra Siberian genetic cline represented by many modern Uralic-speaking populations, and the genetic heterogeneity observed is consistent with the current understanding of the ST being a transcultural phenomenon. Individuals from the second site, Bolshoy Oleni Ostrov in Kola, in comparison form a tighter cluster on the Siberian ancestry cline. We further explore this Siberian ancestry profile and assess the role of the ST phenomenon and other contemporaneous BA cultures in the spread of Uralic languages and Siberian ancestry.

## Abstract (web summary)

This study highlights Bronze Age northern Eurasia as a region of interaction between various groups sharing a Siberian ancestry component and the spread of languages and cultures.

## Introduction

**Fig. 1:**
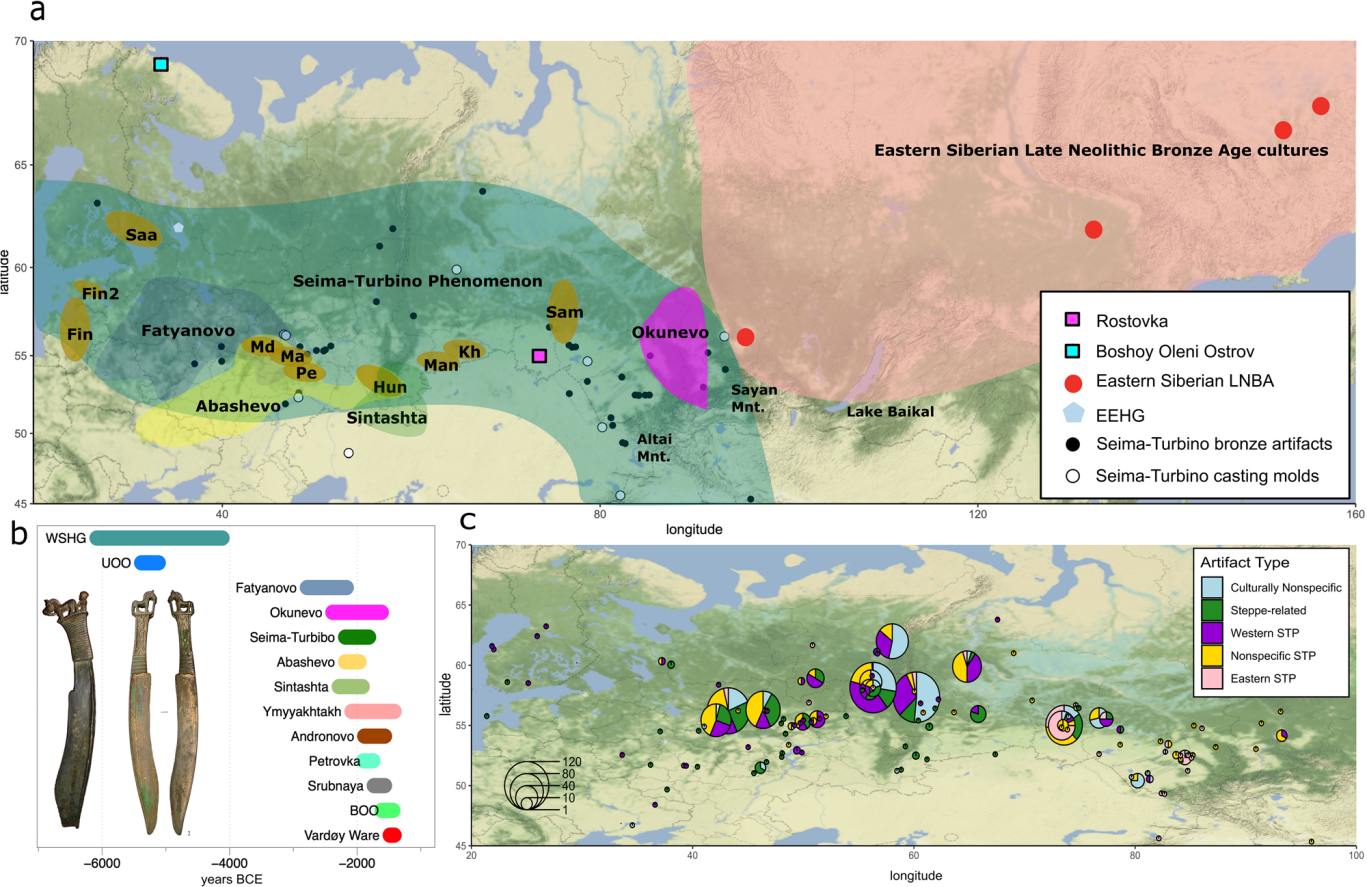
Location of sites, cultures, and language groups mentioned in this study. **a** Geographic map with ROT and BOO indicated, also showing hypothetical origins for ancestral stages of Uralic subfamilies (Saa Saami, Fin/Fin2 Finnic, Man Mansi, Kh Khanty, Sam Samoyedic, Hun Hungarian, Md Mordvin, Ma Mari, Pe Permic), and a distribution of contemporaneous archeological cultures (adapted from Grünthal et al.^8^) , as well as sites with Eastern European Hunter-Gatherer (EEHG) and Eastern Siberian Late Neolithic/Bronze Age (LNBA) individuals. **b** Chronology of Seima-Turbino (ST is including ROT) and BOO individuals together with relevant BA groups of Northern Eurasia. The timeline is based on a combination of absolute (^14^C) and relative dates, **c** Cultural/regional attribution of the metallic inventory of the sites of the ST phenomenon. Pie charts indicate the breakdown of artifacts at specific sites by cultural/regional attribution.

Bronze Age Eurasia (~3000–1000 BCE) is characterized by the intensified development and spread of metallurgy, which had emerged in various regions during the preceding Copper Age and is considered to be one of the most important cultural innovations in human history. The Early Bronze Age in Eurasia (~3000 BCE) is associated with the emergence of the Circumpontic Metallurgical Province, and eastward expansion of metallurgical production and exchange across the Eurasian steppe^1–3^. In the Late Bronze Age (~2200–1000 BCE), a westward movement of materials was also detected, specifically in connection with the so-called Seima-Turbino (henceforth ST) phenomenon^1,2^ characterized by the presence of specific metal artifacts throughout the forest and forest-steppe regions of Northern Eurasia^4^ (Fig. 1). The ST is represented by several sites throughout Eurasia dating to ~2200–1900 BCE and constitutes a “metallurgical network” of many shared traits, such as the use of tin-copper, comparable artifact types, and shared metallurgical technologies that may have involved a movement of craft workers or groups^4,5^. The ST has been described as a “transcultural” phenomenon, i.e., a network of metallurgical production with shared traits on top of an underlying basis of pottery types otherwise consistently associated with various archeological cultures throughout northern Eurasia (see Supplementary Note 1 for a more in-depth discussion of the ST phenomenon).

The people buried with ST-objects have been archeologically described as metallurgists who developed elaborate and distinct bronze objects, and possibly used river systems for transportation^4^. Even though the horse plays a central role in the ST iconography, it remains unclear whether people associated with the phenomenon were using horses for riding, traction or transport. It has been hypothesized that the number of people associated with the ST phenomenon was small, since there are very few sites with human burials linked to the phenomenon, and ST metal artifacts are comparably few but geographically widespread. The early history of the ST phenomenon is not well understood, however, based on the presence of tin and copper in metal alloys of ST objects and the earliest examples of bronze weaponry of the ST-type, the Altai and Sayan mountains have been proposed as geographical origins^4,6,7^. In the entire spatial distribution of the ST, there is a certain degree of regional variation, resulting in a distinction of eastern ST, western ST, non-specific ST, steppe-related, and general culturally non-specific artifacts (see Supplementary Note 1, Fig. 1c, Supplementary Figs. 1–3).

A recent study in Uralic historical linguistics by Grünthal et al.^8^ suggests that the people associated with the ST phenomenon were involved in the westward spread of the Uralic languages^8–10^. The authors propose that Proto-Uralic dissociated rapidly into ancestral sub-branches ~4000 years ago^8^, which overlaps with the dating of the ST transcultural phenomenon^11^. The geographic distribution of the assumed speaker areas of ancestral Uralic subbranches^12^ also co-occurs with ST sites. This hypothesis is further supported by the earliest presence of ST-like artifacts in the Baikal-Sayan area, one of the proposed distribution areas of proto-Uralic speakers^7,13,14^. Lastly, modern speakers of the Uralic language family are characterized by the presence of the Siberian ancestry, which is also present in the individuals from the Bolshoy Oleni Ostrov^15–17^, who we further analyze together with the ST individuals in this study.

The Siberian ancestry component shared by the modern-day speakers of the Uralic language family^15–17^ has been hypothesized to have spread to Europe via the ancient Uralic speakers. This component is present in the genetic profiles of Finnish, Estonian, Saami-speaking individuals, and indigenous Siberian populations today^16^. A previous ancient DNA (aDNA) study focusing on the Eastern Baltic found a genetic contribution from Siberia in the Iron Age, which was linked to the time of the arrival of Uralic languages to the region^15^. However, the Y-haplogroup N1a1a1a1a (previously known as N3a), which is primarily found in present-day northern Eurasian and Uralic speaking groups, first appears in Europe in the Bronze Age individuals from the Bolshoy Oleni Ostrov site (BOO)^17^, in northwestern Russia, together with evidence of high levels of genome-wide Siberian ancestry^17^. Direct or indirect contacts between BOO and southern and western Scandinavia have been proposed based on the archeological record^17–19^, but BOO has not been associated with any known Bronze Age cultures. The presence of ‘Waffle’ Ware ceramics in the archeological record of BOO showing similarities to Neolithic pottery from Yakutia and Chukotka^18^ led archeologists to hypothesize a westward migration of Siberian populations along the forest-tundra or forest-steppe zones. Another ancestry component shared among the populations of northern Eurasia is the Ancient North Eurasian (ANE) ancestry, also known as Upper Paleolithic Siberian ancestry, first described in individuals from Mal’ta and Afontova Gora 2 and 3^20,21^.

Here, we present ancient human DNA data from a well-known, ST-associated burial site Rostovka (ROT) (ca. 2200–2000 cal. BCE), which is one of the very few ST sites with preserved human remains (Supplementary Note 2). The majority of the graves found at Rostovka contain bronze ST objects, bronze weapons and tools, casting molds, jewelry, bone knife handles, and armor plates^11^(Supplementary Note 3, Fig. 1b). To investigate the demographic history of Northern Eurasia, especially in the context of the Siberian genetic component and ANE, we also present new genome-wide data from two new BOO individuals and shotgun data for five published individuals (including one high coverage genome of 40×).

We report the results of joint population genetic analyses of both sites in comparison with published ancient data from chronologically, geographically, and archeologically relevant cultures of the forest-tundra (taiga and tundra) and forest-steppe zones of Eurasia. Together, we aim to provide an updated view on the genetic history and connections of populations of the forest-tundra-steppe and western Siberia, with an emphasis on the ST phenomenon in the context of metallurgical production and a potential link between the spread of the Siberian ancestry and Uralic languages.

## Results

We report genome-wide SNP data for nine individuals from the ST site Rostovka, new data for two BOO individuals, and shotgun genome data for five already published BOO individuals (Table 1). We performed 1240k SNP^22,23^ and mitochondrial genome captures on the nine individuals from ROT, and the two new BOO individuals, as well as Y-chromosomal capture^24^ on just the males. Lastly, we generated shotgun sequence data for five published BOO individuals, including one 40× covered individual (Fig. 1a, Table 1, Supplementary Data 1). Of the newly analyzed individuals, eight ROT individuals were genetically male and one was female, while both new BOO individuals were female. Biological relatedness among the newly reported individuals was estimated using READ^25^, Pairwise Mismatch Rate (PMR), KIN^26^, and lcMLkin^27^ (Supplementary Data 2–5). Based on consistent results across these analyses, we identified a pair of second-degree relatives (ROT011 and ROT015), both of whom are males carrying Y-haplogroup C2a, and could either represent a grandson/grandparent, a nephew/uncle pair or paternal half-siblings, consistent with overlapping radiocarbon dates for both individuals (Table 1). A second-degree related pair was also found among the BOO individuals (BOO004-BOO005).

**Table 1:** General overview of the ROT and BOO individuals included in the study

Sample	Gen. sex	1240k SNPs	Shotgun coverage	Y hg	Y hg terminal SNP	MT hg	Date
ROT002	XY	211,602	–	N1a	L392	G2a1	1938-1700 calBC (±2σ)^a^
ROT003	XY	40,320	–	R1a	M417	R1a1a	ca. 2200-1850 BC, stratigraphic context
ROT004	XY	194,796	–	Q1b	M346	H1	2202-1983 calBC (±2σ)^a^
ROT006	XY	33,591	–	R1b	M73	A10	ca. 2200-1850 BC, stratigraphic context
ROT011	XY	44,705	–	C2a	L1373	C4	2054-1774 calBC (±2σ)^a^
ROT013	XX	25,543	–	–	–	R1b1	ca. 2200-1850 BC, stratigraphic context
ROT015	XY	116,594	–	C2a	F9992	C1e	2133-1919 calBC (±2σ)^a^
ROT016	XY	257,502	–	R1a	Z645	U5a1 + @16192	2137-1919 calBC (±2σ)^a^
ROT017	XY	306,179	–	Q1b	L53	H101	ca. 2200-1850 BC, stratigraphic context
BOO001	XX	–	3.1×	–	–	U4a1^c^	–
BOO003	XX	–	2.3×	–	–	T2d1b1^c^	–
BOO004	XY	–	40×	N1a	L392	C4b^c^	1735-1538 calBC (±2σ)
BOO005	XX	–	4.8×	–	–	U5a1d^c^	–
BOO006	XX	–	2.5×	–	–	D4e4^c^	–
BOO008	XX	696,673	–	–	–	Z1a1a	–
BOO009	XX	814,966	–	–	–	U5a2	992-813 calBC (±2σ)^b^ from charcoal

*Gen. sex* genetic sex, *1240k SNPs* SNP coverage on the 1240k array, *Y hg* Y chromosome haplogroup, *MT hg* mtDNA haplogroup.

^a^From^11^.

^b^From^18^.

^c^From^17^.

We generated a radiocarbon date for individual BOO004, whose genome was shotgun sequenced to 40× coverage (Table 1). The radiocarbon date (MAMS-57646) was determined to be 3351 ± 25 BP, or 1735-1538 calBC (±2σ) after calibration with OxCal 4.4^28^, and 1504-1220 calBC (±2σ) when correcting for a potential freshwater reservoir effect using the Marine 20 curve^28^. The corrected date is an approximation because we do not know the extent of fish consumption in the BOO individuals.

### General population genetic analyses

**Fig. 2:**
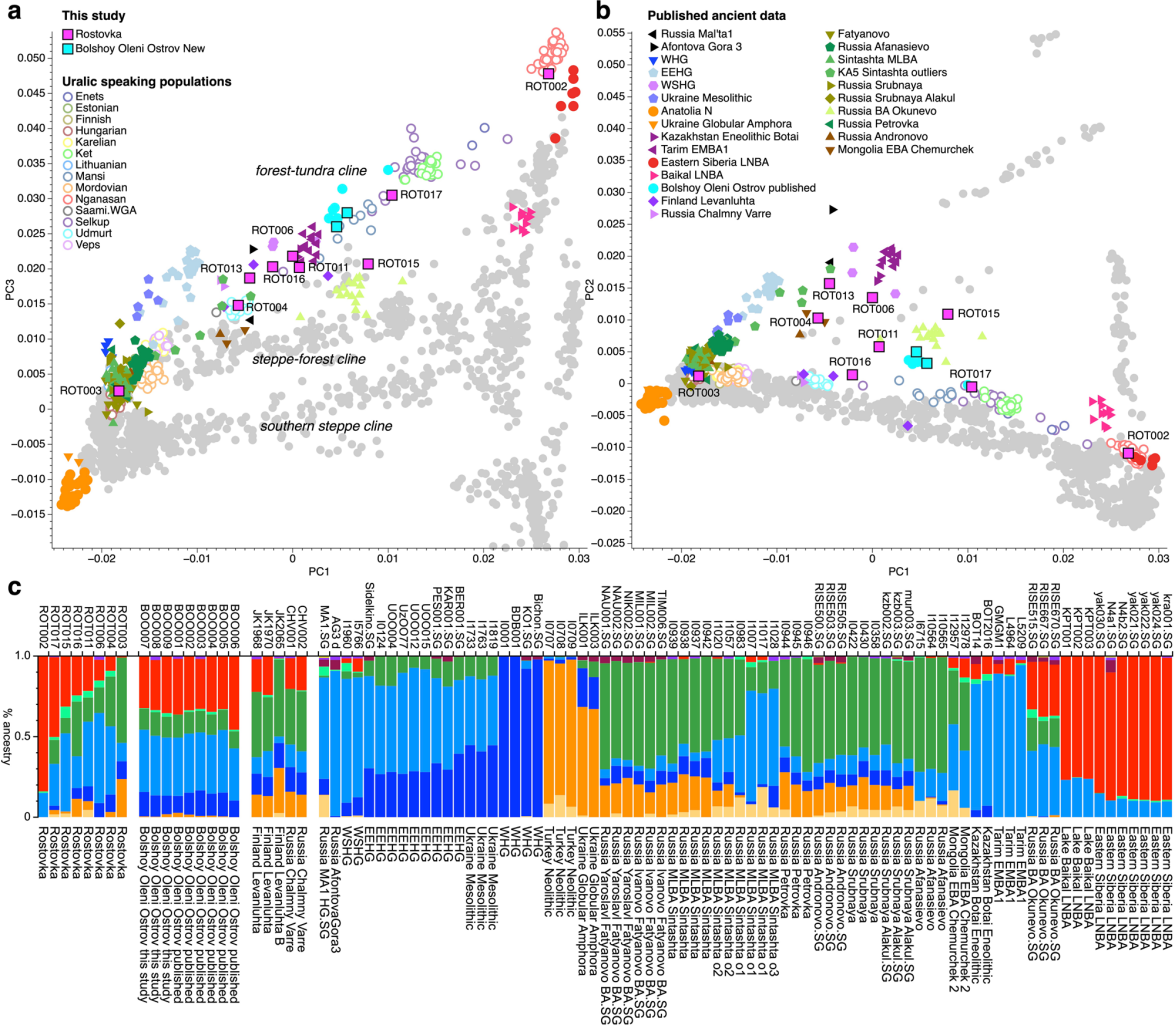
Population structure of ancient North Eurasians. **a** Principal component analysis plot with newly typed (colored symbols with black outline) and published (no outline) ancient individuals projected onto modern variation calculated using modern Eurasian and North American populations from AADR v44.3^71^. Modern populations are shown as gray circles and modern Uralic speaking groups as open circles. Ancient reference individuals are listed under “Published ancient data”, and the new individuals are listed under “This study”. PC1 vs PC3 are plotted, which reveals three genetic clines (labeled in *Italics*) between Western and Eastern Eurasian populations; **b** PCA results for PC1 vs PC2; **c** Unsupervised ADMIXTURE results (k = 10) of a representative subset of the relevant populations and sample names shown in the PCA plot. WSHG West-Siberian Hunter-Gatherers, EEHG Eastern European Hunter-Gatherers, WHG Western Hunter-Gatherers, LNBA Late Neolithic/Bronze Age, MLBA Middle/Late Bronze Age.

We used smartPCA^29^ to perform a principal component analysis (PCA) of modern-day reference populations from Eurasia and the Americas, onto which the ROT and the BOO individuals were projected (Fig. 2a, b). When assessing the genetic structure of Eurasian populations, plotting PC1 vs. PC2 (Fig. 2b) allows us to separate west and east Eurasian populations from the Native American groups, while plotting PC1 vs PC3 (Fig. 2a) distinguishes the major Eurasian ecological zones^30,31^. When plotting PC1 vs PC2, the ANE ancestry cline becomes apparent including individuals from Afontova Gora, Mal’ta1, Botai, West Siberian hunter-gatherers (WSHG), and others. ROT individuals vary along the ANE ancestry cline, while the BOO form a tight cluster within the variation seen at ROT. ROT and BOO individuals fall on the Eurasian PCA (PC1 vs PC3), mainly along a genetic cline of present-day populations that occupy the ecological ‘forest-tundra’ zone (after Jeong et al. ^31^; Fig. 2a), which coincides with the distribution of modern-day Uralic speaking groups and represents the Siberian ancestry variation. BOO individuals form a tighter and more homogeneous cluster, in the middle of the cline between Eastern_Siberia_LNBA and the EEHG, that can be seen with both the PCA and the ADMIXTURE analyses, in line with what has been previously reported^17^. By contrast, the ROT individuals are genetically more heterogenous and spread on a triangle (Fig. 2b) between the Western Steppe Middle to Late Bronze Age cluster (e.g. Sintashta_MLBA^32^), Eastern_Siberia_LNBA and WSHG individuals, which is also visible in the results from unsupervised ADMIXTURE (k = 10) (Fig. 2c, Supplementary Fig. 4).

### Uniparentally inherited haplogroups

We performed Y-haplogroup (Y-hg) typing of the ROT males using the YMCA method^24^ (Table 1) and identified two individuals who carry Y-hg R1a (ROT003: R1a-M417 and ROT016: R1a-Z645), one of the most widely distributed Y-hgs in Eurasia^33^. However, both individuals could be R1a-Z645, since ROT003 does not have coverage on either ancestral or derived ISOGG list SNPs downstream of R1a-M417. Generally, due to their geographic distribution, these R1a sub-lineages are thought to represent the eastward movement of Corded Ware-, and Fatyanovo-associated groups. ROT002, the individual with the highest proportion of north Siberian ancestry, was assigned to Y-hg N1a (N-L392). This Y-hg has also been found in two BOO individuals^17^. Lineage N-L392 is one of the most common in present-day Uralic populations which highlights the potential importance of Y-hg N-L392 in the dissemination of proto-Uralic. One of the male individuals (ROT004) was assigned to haplogroup Q1b (Q-M346), which is found throughout Asia, including in several Turkic speaking populations, e.g.,Tuvinians, Todjins, Altaians, Sojots, and the Mongolian-speaking Kalmyk population^34^. ROT017 carries Y-hg Q1b (Q-L53), which is also common among present-day Turkic speakers across Eurasia. The branch Q-YP4004 includes Central Asian Q-L53(xL54) lineages and one ancient Native American individual from Lovelock Cave in Nevada^35^, while the oldest Q-L53 individual is irk040 (Baikal Neolithic, 4846 BP)^36^. The lineage C2a-L1373, carried by ROT011, is found at high frequency in Central Asian populations, North Asia and the Americas. Lastly, ROT006 carries Y-hg R1b (R1b-M73), a sister-clade of R1b-M269, which is common in the Caucasus, Siberia, Mongolia, and Central Asia today^34^. Overall, the Y-hg lineage diversity of male ROT individuals is consistent with the heterogeneous nature of the ST^37^.

We also identified a large diversity in the mitochondrial haplogroups (mt-hg) among ROT (Table 1), including mt-hgs that are found commonly in east Eurasia (A10, C1, C4, G2a1)^38–41^ and in west Eurasia (H1, H101, U5a, R1b, R1a)^42,43^. Consistently, the individual ROT002 with the highest affinity to Siberia_LNBA and carrying the Y-hg N-L392 also carries a mt-hg G2a1 commonly found in Eastern Eurasia. Analogously, individual ROT003 who carries Sintashta_MLBA-like ancestry and the Corded Ware-derived Y-hg R1a1a1, is also a carrier of the R1a1a mt-hg commonly found in west Eurasia.

### F-statistics

We used F-statistics^44^ to formally assess the relationship of the ROT and BOO individuals with each other, and with different modern and ancient reference individuals and populations. First, we performed outgroup *f*_3_-statistics of the form *f*_3_(Mbuti; test, modern) to test for the affinity of each ROT and BOO individual with modern world-wide populations (Supplementary Fig. 5, Supplementary Data 6). The *f*_3_-statistics results mirror the distribution of the samples in the PCA and ADMIXTURE analyses, wherein the individuals with higher proportions of Eastern_Siberia_LNBA ancestry (e.g. ROT002) show a greater affinity to modern-day Siberian and Uralic-speaking populations, such as Nganasan, Evenk, Negidal, Nanai, and Ulchi (Supplementary Fig. 5A), whereas the individuals with more Sintashta-like Western_Steppe_MLBA ancestry (e.g., ROT003) are closer to modern-day (North) Europeans, including Norwegian, Belarusian, Lithuanian, Scottish and Icelandic individuals (Supplementary Fig. 5B). Comparisons with ancient groups/individuals using *f*_3_(Mbuti; test, ancient) showed a similar trend (Supplementary Fig. 5). ROT002 on the ‘eastern end’ of the Eurasian cline shares more genetic drift with Eastern_Siberia_LNBA, Russia Ust Belaya Neolithic, and Mongolia Early Iron Age individuals (Supplementary Fig. 5A). By contrast, ROT003, the ‘westernmost’ individual in the Eurasian PCA space, has the highest affinity to Lithuania early Middle Neolithic Narva, Russia Sintashta, Kazakhstan Georgievsky Middle Bronze Age, Russia Poltavka, and Serbia Mesolithic individuals (Supplementary Fig. 5B). Similar trends can be observed for BOO, wherein the modern Uralic-speaking populations, such as Nganasan and Selkup, are among the tests with the highest *f*_3_- statistics. The ancient individuals most closely related to BOO are EEHG, WSHG, Botai and Tarim Early/Middle Bronze Age (EMBA) individuals carrying high levels of ANE ancestry (Supplementary Fig. 5J–R).

**Fig. 3:**
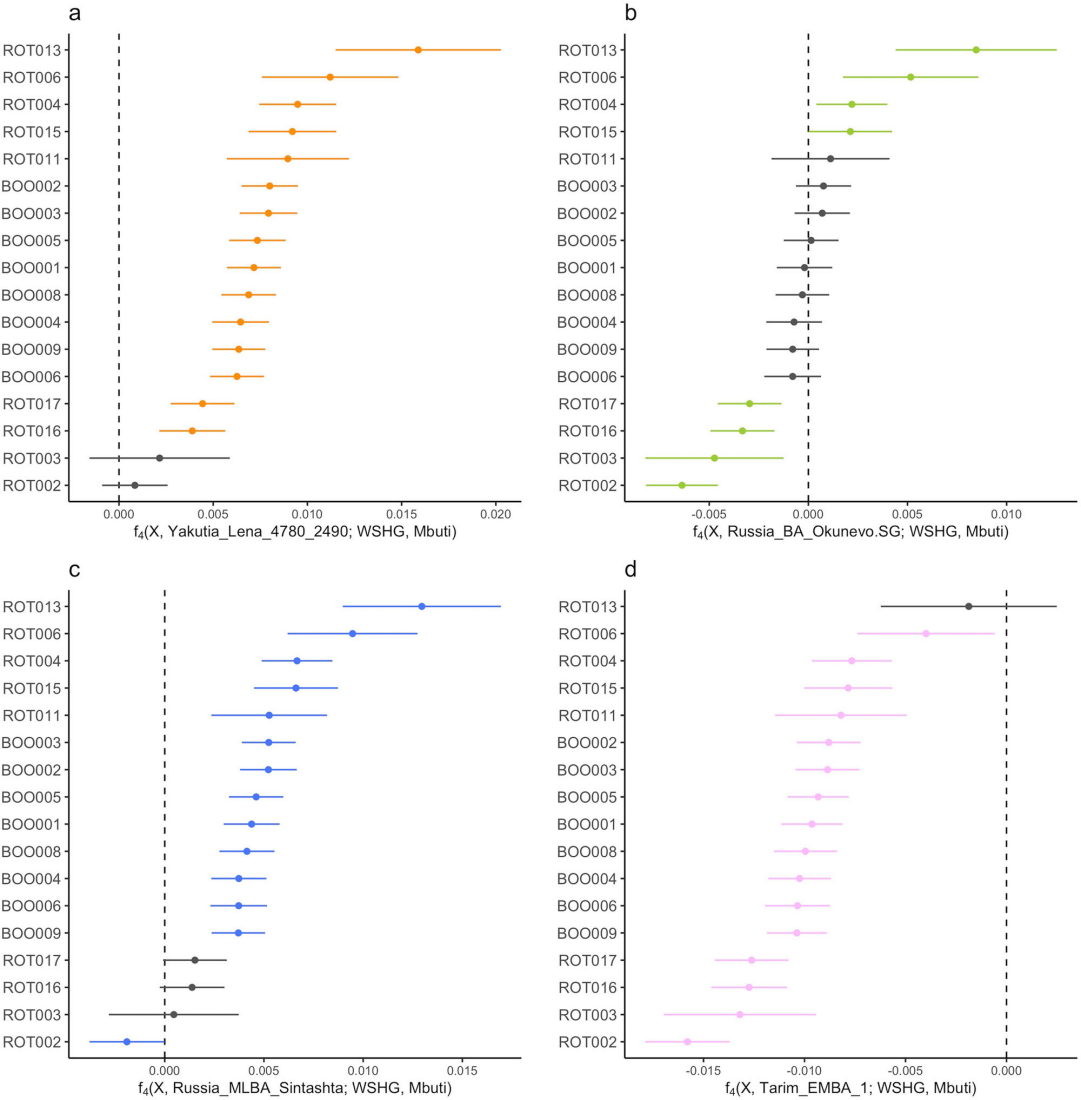
Genetic affinities between North Eurasian populations. *f*_4_-statistics testing for excess WSHG ancestry in ROT and BOO individuals with respect to **a** Yakutia Lena 4780-2490 (Siberia_LNBA), **b** Okunevo, **c** Russia MLBA Sintashta, and **d** Tarim EMBA1. Significantly non-zero *f*_4_-statistics (|Z| > 3) are shown in color, and non-significant *f*_4_-statistics are shown in gray. All error bars indicate 3 standard errors. “X” denotes the individuals given on the y-axis.

Based on the geographic location of the sites, we tested whether ROT and BOO individuals retained more local ANE ancestry compared to contemporaneous groups from similar general geographic area, time period, and archeological affiliation, using *f*_4_-statistics of the form *f*_4_(X, *test*; WSHG, Mbuti) where X stands for ROT and BOO individuals, and *test* populations include Okunevo, Tarim_EMBA_1, Sintashta_MLBA, and Eastern_Siberia_LNBA (Fig. 3). This test allowed us to identify groups that form a clade with ROT and BOO, and cases where ROT and BOO may have additional affinity to ANE ancestry represented here by WSHG from Russia as the best spatial and temporal proxy. We find that ROT and BOO individuals carry excess affinity to ANE when compared to Eastern_Siberia_LNBA (Fig. 3a) and Russia MLBA Sintashta (Fig. 3c), except for ROT002 and ROT003. All BOO individuals are symmetrically related to the Okunevo Bronze Age group indicating no additional affinity to ANE (Fig. 3b). However, we see more heterogeneity in ROT, with some individuals having significantly more, and others significantly less genetic affinity to WSHG compared to Okunevo (Fig. 3b). All but one individual (ROT013) have significantly less ANE ancestry compared to Tarim EMBA (Fig. 3d). The general observations from *f*_4_-statistics formally confirm the PCA results (Fig. 2), where ROT individuals vary in their location with regards to WSHG, i.e., ANE ancestry affinity, while the BOO individuals are more homogeneous.

The genetic profile of BOO individuals is intriguing, when compared to present-day individuals of the same geographic area of Scandinavia and western Russia (Fig. 2). However, the cultural affiliation of the BOO individuals remains poorly understood. Based on pairwise outgroup-*f*_3_-statistics with different ancient populations from Scandinavia, Anatolia_N, and Sintashta_MLBA, the BOO and ROT individuals separate from the rest of the ancient populations (Supplementary Fig. 6). The *f*_3_- and *f*_4_-statistics together show a non-local genetic origin for the BOO individuals, with no substantial levels of early European farmer ancestry, which thus excludes contact with contemporary and genetic contribution towards subsequent Scandinavian groups.

### qpAdm modeling

**Fig. 4:**
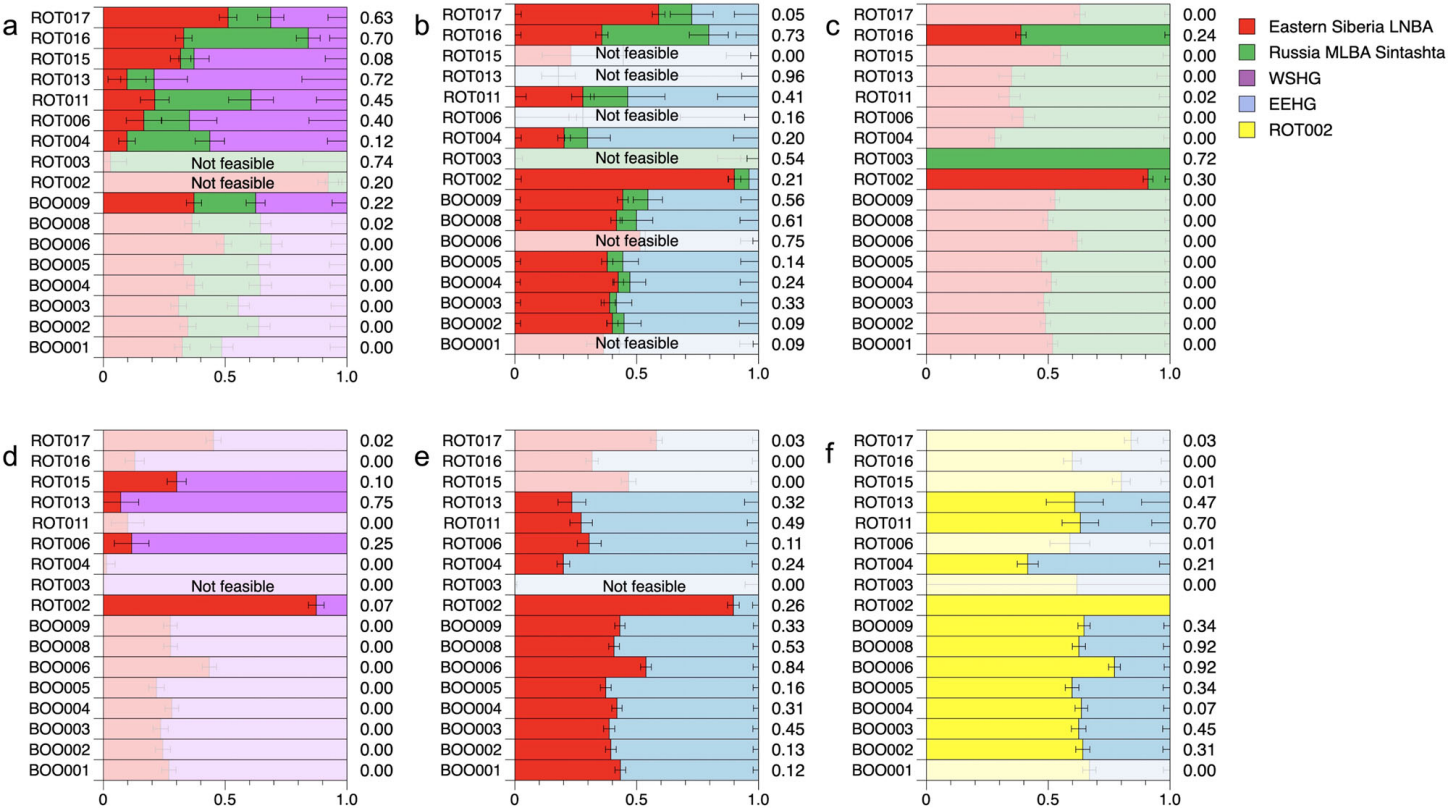
Ancestry modeling for ROT and BOO individuals with qpAdm. **a** qpAdm models using Eastern Siberia LNBA, Russia MLBA Sintashta, and WSHG as sources; **b** Models with Eastern Siberia LNBA and Sintashta as sources; **c** Models with Eastern Siberia LNBA and WSHG as sources; **d** Models with Eastern Siberia LNBA and EEHG as sources; **e** Models with Eastern Siberia LNBA and EEHG; **f** Models with ROT002 and EEHG. Corresponding *p*-values for each analysis are shown to the right of each row. Models with *p*-values < 0.05 are grayed out, and the models with negative ancestry proportions are indicated as “Not feasible”.

Lastly, we performed qpAdm analysis to formally test for and quantify the admixture proportions of potential source populations for ROT and BOO individuals (Fig. 4, Supplementary Data 7). Here, we successfully modeled the ROT individuals as a mix of three sources (Eastern_Siberia_LNBA, Sintashta_MLBA, and WSHG), except for ROT002, which we modeled instead as a two-source mixture of mainly Eastern_Siberia_LNBA ancestry and a smaller proportion of EEHG-like ancestry that could be represented by either Sintashta_MLBA, WSHG, or EEHG, and ROT003 which we modeled with Sintashta_MLBA as single source (Fig. 4b). We also tested whether ROT individuals could be modeled as a two-way mixture of the Eastern_Siberia_LNBA ancestry and either Sintashta_MLBA or WSHG as sources, however, this combination of ancestries did not result in consistently plausible model fits, compared to the combination of all three ancestries (Fig. 4a–c). By contrast, BOO individuals could not be modeled using either the combination of all three ancestry sources (Eastern_Siberia_LNBA, Sintashta_MLBA, and WSHG), or just a two-way mixture (Fig. 4a, c, Supplementary Data 7). However, replacing WSHG with EEHG as the putative local hunter-gatherer ancestry substrate and using Eastern_Siberia_LNBA as a second source provided good model fits (Fig. 4d, Supplementary Data 8). Importantly, all BOO individuals, except for BOO001, could also be modeled as a mixture of ROT002 and EEHG (Fig. 4e, f, Supplementary Data 8) suggesting, together with the results from the outgroup f_3_-statistics (Supplementary Fig. 6), that BOO individuals may represent a subset of the diversity present in ROT.

### Identity-by-descent (IBD) analysis

To investigate distant biological relatedness among the BOO individuals, we first imputed the genomes using GLIMPSE^45^ with the 1000 G dataset^46^ as a reference panel (ROT individuals are below the required coverage threshold for imputation). Based on the identification of haplotype blocks of certain lengths that are shared between individuals, i.e. identical by descent^47^, we confirmed the above identified 2^nd^-degree related pair (BOO004-BOO005), and also found two third-degree related pairs (BOO003-BOO004 and BOO003-BOO005), as well as multiple pairs potentially related in the fourth-fifth-degree (Supplementary Data 9). The observation that the BOO individuals are distantly related to each other explains the relative homogeneity seen in the sample compared to ROT. According to the archeological context, two pairs of biologically related individuals were buried in the same grave: third-degree related pair BOO003 (burial 16, sepulture 1, female) and BOO004 (burial 16, sepulture 3, male); and one 4th/5th-degree related pair BOO005 (burial 17, sepulture 3, female) and BOO009 (burial 17, sepulture 4, female)^18^.

**Fig. 5:**
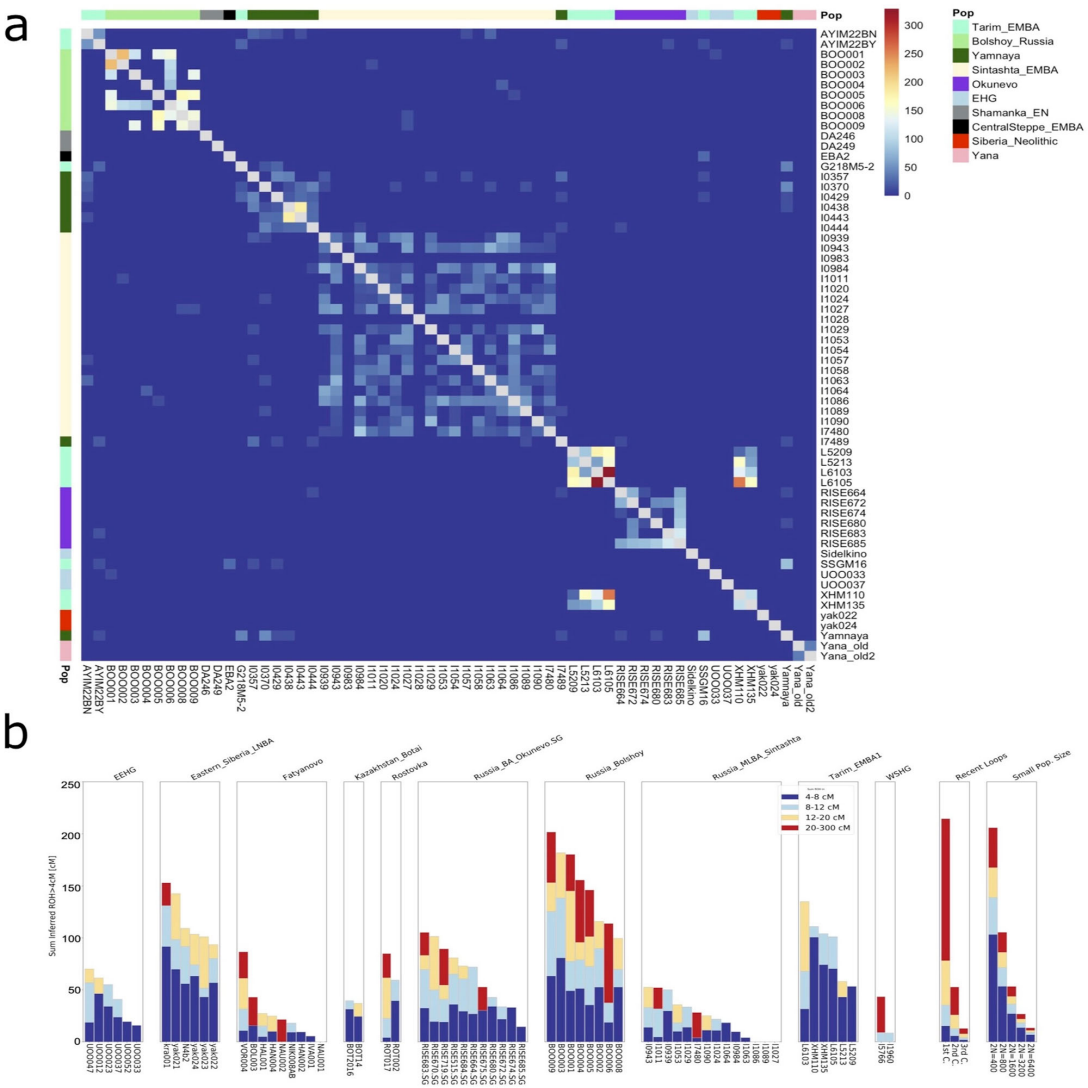
Estimation of shared identity-by-descent and parental background relatedness. **a** IBD sharing between BOO and published data. Shared IBD chunks between 12 and 30 cM in length are shown. The total IBD length shared is indicated by the color of the square, and population designation is shown on the y-axis. **b** HapROH output for BOO, ROT and relevant contemporaneous populations. Runs of homozygosity (ROH) are plotted by population for individuals with more than 400k SNPs on the 1240k panel. ROH segments are colored according to their binned lengths.

We also tested for IBD sharing between BOO and published individuals who are broadly contemporaneous and geographically close, including Tarim_EMBA^48^, Okunevo^42^, Sintashta_MLBA^32^, EEHG^49^, Botai^42^, Yamnaya^42^, Easter_Siberia_LNBA^36^, and others (Fig. 5a, Supplementary Data 9). We found three shared IBD fragments (14–22 cM) between BOO individuals and Sintashta_MLBA individuals (Supplementary Data 9), potentially suggesting shared ancestors as recent as approximately 500–750 years, and most likely reflecting the shared EEHG ancestry that is present in both groups.

### Runs of homozygosity

To investigate the underlying population structure, general parental background relatedness, and effective population sizes, we used HapROH^50^ to analyze runs-of-homozygosity (ROH) in the genomes of the BOO individuals together with a set of published individuals with more than 400k SNPs on the 1240k panel. We compared BOO to geographically and genetically close individuals from the Eurasian forest-tundra-steppe area, associated with Okunevo, Sintashta_MLBA, EEHG (UOO), Eastern_Siberia_LNBA, Tarim EMBA, and Fatyanovo cultures (Fig. 5b). We also included two ROT individuals with more than 200k SNPs, but these results should be interpreted with caution. The ROH results of BOO individuals suggests that this early Metal Age group had a relatively small effective population size of ~2 N = 800, and one of the individuals (BOO006) appears to be an offspring of second cousins. Tarim EBMA, Okunevo, and Eastern_Siberia_LNBA groups also seemed to have relatively small effective population sizes, while Fatyanovo and Sintashta-associated groups potentially had larger effective population sizes (Fig. 5b). In comparison, ROT individuals show similar ROH profiles to the populations they are closely related to, based on the PCA and *F*-statistics, i.e., ROT002 resembles the Eastern Siberian LNBA, and ROT017 the BOO individuals (Fig. 5b).

### Demographic modeling

High-coverage shotgun data from BOO004 allowed us to perform demographic modeling to investigate North Eurasian genetic ancestry and the nature of the admixture of the Eastern and Western Eurasian sources found in BOO individuals using a site-frequency spectrum (SFS) modeling-based method called momi2^51^. We included published data from representative North Eurasian populations, both preceding and contemporaneous to BOO. We also used DATES v.753^52^ to estimate the date of the admixture event in BOO individuals between the EEHG and Eastern_Siberia_LNBA sources to be 17.98 ± 1.06 generations ago, or around 500 calendar years prior to the mean radiocarbon date of BOO, assuming a generation time of 29 years^53^ (Supplementary Fig. 7). This results in an approximate date of admixture ~4086 or ~3800 years ago when the marine reservoir correction is taken into account.

**Fig. 6:**
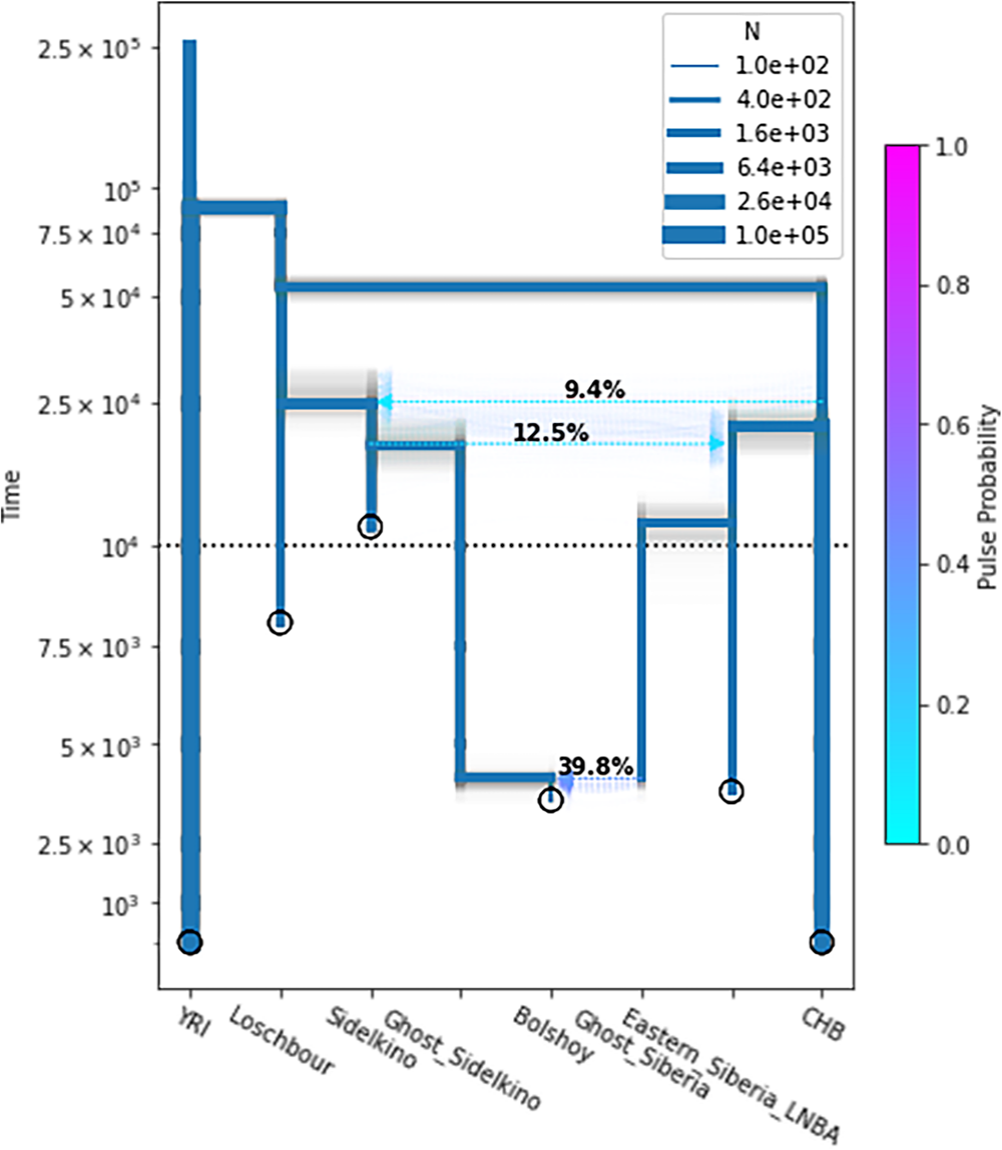
Demographic modeling of North Eurasian populations. Momi2 demographic model for BOO004 using shotgun sequencing data from published ancient and modern individuals. Point estimates of the final model are shown in blue; results for 100 nonparametric bootstraps are shown in gray. The sampling times of populations are indicated by circles and population size estimates by the thickness of branches. The y-axis is linear below 10,000 years ago, and logarithmic above it. See Supplementary Data 10 for specific parameter values. YRI Yoruban, CHB Han Chinese.

After an incremental build-up of our momi2 model (Supplementary Note 4, Supplementary Data 10–12, Supplementary Tables 1–6, Supplementary Figs. 8–12) and including three admixture events, our final model estimated the split times between Africans (Yoruba, YRI) and Eurasians (Loschbour) 87,790 years ago (95% CI 85,250–91,040), and between Western Eurasians (Loschbour) and Eastern Eurasians (CHB) at 53,010 years ago (95% CI 49,200–55,540). The divergence between the lineage leading to the Eastern Siberia LNBA and CHB was found to be 21,580 years ago (95% CI 18,600–24,810). We then modeled gene flow from the lineage leading to CHB to the EEHG at 9.4% (95% CI 4.4%–14.7%). The effective population size N_e_ for Eastern Siberia LNBA was found to be 1690 (95% CI 1380–2020), and the population size for EEHG - 2470 (95% 1930–3790). The gene flow event from EEHG to East Siberian LNBA was modeled at 12.5% (95% CI 7.77%–15.7%). These gene flow events are in line with the shared ANE ancestry history in both lineages. We estimated a recent admixture for BOO individuals (95% confidence interval (CI) 3778–4357 years ago), with substantial gene flow (39.8%; 95% CI 34.9–44.4%) from Eastern Eurasians (represented here by Eastern Siberia LNBA). Importantly, the mixture proportions are consistent with the results from qpAdm, and the date estimates overlap with those from DATES. The population size estimated for BOO (N_e_ = 235, 95% CI 118–441) from momi2 (Fig. 6, Supplementary Data 10) is at the smaller end of the estimate obtained from hapROH (2 N between 400 and 800 individuals, Fig. 6), which is likely an effect of momi2 not taking into account inbreeding via the analysis of the runs of homozygosity.

## Discussion

The production of metals is an important human cultural innovation that has developed several times in multiple regions around the globe. In Bronze Age Northern Eurasia, the Seima-Turbino transcultural phenomenon exemplifies this innovation horizon based on the evidence of skilled metallurgical production, which is visible in the archeological record of many sites across a vast geographic area.

In this study, we analyzed genome-wide data of ST-associated individuals and their connections to contemporaneous BA and preceding archeological groups of the northern Eurasian forest-tundra-steppe zone, such as Sintashta and Okunevo, as well as Neolithic and BA Siberian groups. In this light, we also reassessed the genetic structure of BOO individuals from Kola Peninsula in northwestern Russia who have been shown to carry high levels of Siberian ancestry, an important characteristic of northern Eurasian populations.

The observed genetic heterogeneity among the ROT individuals can either reflect a group at an early stage of admixture with genetic influences from various regions or signify the heterogeneous nature of the ST complex^4^. The findings from genome-wide autosomal data in PCA, ADMIXTURE and F-statistics are consistent with Y-chromosomal and mitochondrial hg data. Eight males of nine ROT individuals represent both eastern Eurasian and Western Eurasian Y-chromosomal lineages, and eastern and western Eurasian mitochondrial lineages, respectively. In general, the region of the Middle Irtysh around Rostovka can be characterized as a typological melting pot of the western and eastern part of the ST phenomenon mirrored in the genetic data. Together with evidence from the available archeological data^4^, we argue that the individuals buried at ROT more likely represent a variety of genetic and perhaps cultural backgrounds, brought together by the ST metallurgical network.

On an individual level, there is some evidence for a correlation between genetic ancestry of the screened individuals and the cultural/regional attribution of their grave goods. For example, the bone body armor from grave 34 (ROT016) has close parallels to similar pieces found in burial grounds of the Sintashta culture like Sintashta itself or Kamenjy Ambar 5. The rest of the grave goods show local attributes (socket axes) or are typical for the eastern part of the ST phenomenon (hooked lance heads). Grave 8 (ROT004) shows clearly eastern typological attributes (hooked lance head, ceramics). Grave 24 (ROT011) shows a rather western typology based on the dagger blade of type NK-14, but in its proximity, and clearly associated to it, an eastern object like a hooked lance head and two local artifacts (a socket ax type K-32 and a lance head KD-40) were found. The other graves are rather nonspecific due to the limited number of grave goods, such as graves 7 (ROT003) and 10 (ROT006) or show local attributes like grave 5 (ROT002). Thus, from a typological point of view, the inventory of the graves is as much of admixture of western and eastern elements as the genetics profiles of the buried individuals.

We were able to investigate the demographic history of Northern Eurasia, by reconciling and expanding on published deep population divergence models of Eurasia^42^. Importantly, we were also able to seamlessly integrate the shared ANE ancestry as gene flow between Eastern and Western Eurasian via the lineages leading to CHB and Sidelkino ~25 kyr ago, and between the lineages leading to the Eastern Siberian LNBA and Sidelkino ~19 kyr ago, suggesting a shared ANE ancestry substrate. BOO could be modeled as a recent mixture of the Neolithic Siberian and EEHG components approximately ~4400–3600 years ago, which places this event at a similar time as the temporal peak of the ST phenomenon.

Interestingly, despite the geographic location of the burial site on the Kola Peninsula in northwestern Russia, BOO individuals carry higher proportions of ‘eastern’ Siberian ancestry than most ROT individuals. The genetic homogeneity observed in BOO individuals can be explained by the genetic relatedness as shown by IBD sharing and ROH analysis, which is indicative of a relatively small or isolated population.

We also find that BOO and ROT exhibit distinct genetic subtleties regarding the presence of the Early European Farmer ancestry, despite the broad chronological overlap. In general, ROT individuals carry higher levels of Neolithic farmer-derived ancestry, which we are able to model as part of the Sintashta_MLBA ancestry. However, this ancestry is not present in the BOO individuals, who carry HG-related ancestry that is more similar to an older, but local EEHG stratum (as demonstrated for the nearby Yuzhny Oleni Ostrov burial site on Lake Onega, the Republic of Karelia)^21,54^. The lack of European farmer ancestry in BOO, contrary to what has been reported in Lamnidis 2018 (Fig. 4a)^17^, also highlights the natural limits of the farming subsistence practice and the spread of farmer-related ancestry mediated by MBA forest steppe pastoralists into the northernmost parts of Eurasia during this time period. The presence of ANE ancestry further supports the genetic legacy of a basal North Eurasian lineage that was wide-spread and formed the local genetic substrate in Siberia. This ancestry is generally associated with groups falling on the forest tundra genetic cline^31^, and is present in high levels in the Bronze Age Tarim mummies^48^. This Siberian ancestry has not been found west of the Urals until after the BA.

With the new data from ROT, we are able to assess a recent hypothesis which suggests that Uralic languages could have been used within the ST network leading to the initial spread of Uralic languages across the Eurasian forest steppe^8,10,55^. The genomic profiles of the ST-associated ROT individuals indeed fall on an ancestry cline that generally mirrors the genetic distribution of modern-day Uralic-speaking populations of the northernmost forest-tundra (taiga and tundra) ecological zone^31^. However, our findings also show that the ST-associated individuals from Rostovka likely did not originate from a single population but rather represent people from a wide geographical area. Seima-Turbino was a latitudinal phenomenon on the same east-west axis where also the hypothetical homelands of the ancestral Uralic subgroups were positioned^12^. Thus, our genetic results are temporally and geographically consistent with the proposal that the spread of Uralic languages could have been facilitated within the ST network but are neither a clear nor a direct proof. Further ancient human DNA data from northern Eurasia will help elucidate the details of the wider spread of ancient Siberian ancestry and its association with proto-Uralic speaking groups.

Taken together, our findings show that all but one of the carriers of artifacts associated with the ST transcultural phenomenon have genetic similarities to the current taiga-tundra area populations but harbor a diverse mix of western and eastern Eurasian ancestries. However, due to the limited number of individuals studied, we cannot be certain as to what degree the individuals in this study represent the ST phenomenon as a whole. Genetic data from other confidently ST-associated sites will be crucial in providing a comparative analysis of the data^56^. Lastly, we investigate the genetic history of the Siberian ancestry in northern Eurasia and suggest that there were possibly several waves of migration of people carrying the Siberian ancestry component, indicating a complex, and hitherto unappreciated, demographic history of the region.

## Methods

### Ethics declaration – sample provenance

Permissions to study the archeological samples presented were obtained directly from collaboration partners and co-authors of this study: BOO individuals - permissions were acquired from Valery Khartanovich, head of the Anthropology department at Peter the Great Museum of Anthropology and Ethnography (Kunstkamera). ROT individuals - Permissions were acquired from Sergey Kuzminykh, Leading Researcher at the Laboratory of Natural Science Methods in Archeology at the Russian Academy of Sciences and Igor Kovtun, University of Tomsk.

### Sample information

A total of 19 individuals from Rostovka and 8 from Bolshoy Oleni Ostrov were screened for aDNA preservation using shotgun sequencing of 5 M reads, however, only nine ROT individuals passed the 0.1% endogenous DNA cutoff to be further analyzed using capture arrays. The low success rate is explained by the fact that the macroscopic preservation of the skeletal remains was poor in general, and we could only sample random parts of long bones and few teeth, but no petrous bones. All BOO individuals passed the endogenous DNA cutoff for further processing, and the samples with high % endogenous DNA were sent for high-coverage shotgun sequencing.

### DNA extraction and data generation

All aDNA work was done in dedicated clean laboratory facilities of the (former) Max Planck Institute for the Science of Human History (now Max Planck Institute for Geoanthropology) in Jena, Germany following the standard protocols^57^. A minimally invasive sampling method was used for petrous portion of the temporal bone^58^, and for teeth the crown was separated from the root and the inner pulp chamber was drilled out^59^. A modified protocol after Dabney et al.^60,61^. DNA double-stranded libraries were built for BOO samples using a partial uracil-DNA-glycosylase (UDG-half) treatment^62^, and single-stranded libraries were built for ROT^63,64^. All libraries were double-indexed with a unique pair of indices^65^.

First, shotgun libraries were screened for the presence of endogenous DNA by sequencing 5 M reads on an Illumina HiSeq 4000 or NextSeq500 sequencing platform using a single end (1 × 75-base pair (bp) reads) kit, and samples with the aDNA content above 0.1% were captured for the 1240k sites. We also produced mtDNA and Y-haplogroup capture data for the samples included in the study. A set of BOO individuals were shotgun sequenced to high coverage. The nfcore/eager pipeline v.2.3.5^66^ was used to process the samples from fastq files to the deduplicated bam files. The software version information is listed in Supplementary Data 13. Briefly, samples were mapped to the hs37d5 version of the human reference genome using bwa aln with the following parameters: bwa aln -n 0.01 -o 2 -l 16500. Pseudohaploid genotyping calls for the ROT individuals were produced using pileupcaller (https://github.com/stschiff/sequenceTools) with the --singlestrandmode option. We trimmed two base pairs from bam files of BOO individuals from each side of the read, and genotyped the samples to produce pseudohaploid calls with pileupcaller (https://github.com/stschiff/sequenceTools). The aDNA status of the samples was authenticated using MapDamage v2^67^. Contamination from modern sources was determined using a combination of contammix^68^, schmutzi^69^, ANGSD X-chromosome contamination estimate (for males)^70^, and sex determination. READ^25^ and pairwise mismatch rate (PMR) were used to perform biological relatedness analysis. PMRs were calculated from pseudohaploid genotypes of the 1240k panel.

### Population genetics analyses

The projection PCA was done on the subset of the genotyping data (Human Origins array SNPs ~630k) using smartpca^29^ including already published ancient and modern data from the Allen Ancient DNA Resource (AADR) v44.3^71^ using the projection mode, wherein ancient samples were projected upon modern genetic variation. Unsupervised admixture analysis was done on the subset of ~143k ancestry-informative SNPs^72^ on the ROT and the new BOO data together with already published aDNA samples from the AADR v44.3^71^ using ADMIXTURE^73^ for 1–20 K clusters in 5 iterations. Coefficients of variance for each K were compared and the best K level was chosen based on the lowest average CV.

The f-statistics and qpAdm analyses were performed using admixr^74^ on the entire set of 1240k SNPs. The resulting data were plotted using DataGraph v.4.6.1, and R^75^ using the ggplot2 package^76^. For qpAdm, we used Mbuti, Georgia_Kotias.SG, Israel_Natufian_published, Ami, Mixe, Italy_North_Villabruna_HG, and ONG.SG as an outgroup set (based on^17^).

Mitochondrial haplogroups were determined using HaploGrep2^77^ using the data from the mitochondrial capture. Briefly, mitochondrial capture data was mapped to the mitochondrial reference genome NC_012920.1 using circularmapper^78^ and mapping quality threshold of 30. Bam files were then imported into Geneious and a consensus fasta file was produced with the coverage threshold of 5, and Sanger heterozygotes set to >50%. The consensus fasta file was then imported into HaploGrep2. Y-haplogroup data generated using YMCA was used to assign Y-chromosome haplogroups to male ROT individuals following the method described in^24^.

ROH analysis was done using HapROH^50^ on the pseudohaploid data from BOO, together with already published individuals, and only focusing on samples with more than 400k SNPs from the 1240k SNP array.

BOO samples were imputed and phased using GLIMPSE^45^ following the default parameters, and merged with already published data, in order to test for patterns of IBD sharing among the individuals using ancIBD^47^. IBD analyses were restricted to samples covering more than 600 K SNPs with GP > = 0.99 after genotype imputation. IBD results were plotted using the R package pheatmap^79^.

### Demographic modeling

We used DATES^52^ to determine the time of admixture in BOO using Yakutia_Lena and UOO as the two reference sources. Demographic modeling of BOO was then performed using momi2^51^. We progressively added more populations into the model and optimized the model step by step. When optimizing the final model, we got a series of similar likelihood results with recent admixture time and small population size in Bolshoy Oleni Ostrov lineage. We chose the final model whose admixture time matches the conclusion in DATES. We performed 100 nonparametric bootstraps to ensure the stability of the parameters. See Supplementary Note 4 for a detailed description of modeling with momi2.

### Reporting summary

Further information on research design is available in the Nature Portfolio Reporting Summary linked to this article.

### Supplementary information

Supplementary Information

Supplementary Data

Reporting summary

## Data Availability

Genomic data (BAM and fastq formats) are available on the European Nucleotide Archive (ENA) under accession number PRJEB74730, genotypes in eigenstrat format can be found at https://edmond.mpdl.mpg.de. The momi2 code was uploaded to the Edmond Max Planck repository 10.17617/3.NPAC3S. The source data behind the graphs in the paper can be found in Supplementary Data 6-12.
