# Supplementary material for: Bronze age Northern Eurasian genetics in the context of development of metallurgy and Siberian ancestry
Source: Commun Biol. 2024 Jun 11;7:723. doi: 10.1038/s42003-024-06343-x (PMC11166947; doi:10.1038/s42003-024-06343-x)
Supplement: Supplementary file 3 — Reporting summary [file 42003_2024_6343_MOESM3_ESM.pdf]

nature portfolio

Corresponding author(s): Ainash Childebayeva

Last updated by author(s): Apr 3, 2024

Reporting Summary

Nature Portfolio wishes to improve the reproducibility of the work that we publish. This form provides structure for consistency and transparency in reporting. For further information on Nature Portfolio policies, see our Editorial Policies and the Editorial Policy Checklist.

Statistics

For all statistical analyses, confirm that the following items are present in the figure legend, table legend, main text, or Methods section.

n/a	Confirmed	
☐	☒	The exact sample size (*n*) for each experimental group/condition, given as a discrete number and unit of measurement
☐	☒	A statement on whether measurements were taken from distinct samples or whether the same sample was measured repeatedly
☒	☐	The statistical test(s) used AND whether they are one- or two-sided<br>*Only common tests should be described solely by name; describe more complex techniques in the Methods section.*
☒	☐	A description of all covariates tested
☒	☐	A description of any assumptions or corrections, such as tests of normality and adjustment for multiple comparisons
☒	☐	A full description of the statistical parameters including central tendency (e.g. means) or other basic estimates (e.g. regression coefficient) AND variation (e.g. standard deviation) or associated estimates of uncertainty (e.g. confidence intervals)
☐	☒	For null hypothesis testing, the test statistic (e.g. *F*, *t*, *r*) with confidence intervals, effect sizes, degrees of freedom and *P* value noted<br>*Give P values as exact values whenever suitable.*
☒	☐	For Bayesian analysis, information on the choice of priors and Markov chain Monte Carlo settings
☒	☐	For hierarchical and complex designs, identification of the appropriate level for tests and full reporting of outcomes
☒	☐	Estimates of effect sizes (e.g. Cohen's *d*, Pearson's *r*), indicating how they were calculated

Our web collection on statistics for biologists contains articles on many of the points above.

Software and code

Policy information about availability of computer code

Data collection	No software/code was used for data collection.
Data analysis	Momi2 code used for demographic modeling is shared (see Data Availability). nf-core/eager v2.3.5<br>Nextflow v20.10.0<br>FastQC v0.11.9<br>MultiQC<br>AdapterRemoval v2.3.1<br>fastP v0.20.1<br>BWA v0.7.17-r1188<br>Samtools v1.9<br>endorS.py v0.4<br>Picard MarkDuplicates v2.22.9<br>Qualimap v2.2.2-dev<br>Preseq v2.0.3<br>freebayes v1.3.2-dirty<br>sequenceTools v1.4.0.5<br>MTNucRatioCalculator v0.7<br>bedtools v2.29.2<br>bamUtil v1.0.14<br>pmdtools v0.50<br>angsd v0.933

sexdeterrmine v1.1.2
eigenstrat_snp_coverage
mapDamage2 v2.2.0

For manuscripts utilizing custom algorithms or software that are central to the research but not yet described in published literature, software must be made available to editors and reviewers. We strongly encourage code deposition in a community repository (e.g. GitHub). See the Nature Portfolio guidelines for submitting code & software for further information.

Data

Policy information about availability of data

All manuscripts must include a data availability statement. This statement should provide the following information, where applicable:

- Accession codes, unique identifiers, or web links for publicly available datasets
- A description of any restrictions on data availability
- For clinical datasets or third party data, please ensure that the statement adheres to our policy

Genomic data (BAM and fastq formats) are available on the European Nucleotide Archive (ENA) under accession number PRJEBXXX, genotypes in eigenstrat format can be found at https://edmond.mpdl.mpg.de. The momi2 code was uploaded to the Edmond Max Planck repository https://doi.org/10.17617/3.NPAC3S.

Human research participants

Policy information about studies involving human research participants and Sex and Gender in Research.

Reporting on sex and gender	No biological material or data from human participants was used.
Population characteristics	No biological material or data from human participants was used.
Recruitment	No biological material or data from human participants was used.
Ethics oversight	No biological material or data from human participants was used.

Note that full information on the approval of the study protocol must also be provided in the manuscript.

Field-specific reporting

Please select the one below that is the best fit for your research. If you are not sure, read the appropriate sections before making your selection.

☒ Life sciences ☐ Behavioural & social sciences ☐ Ecological, evolutionary & environmental sciences

For a reference copy of the document with all sections, see nature.com/documents/nr-reporting-summary-flat.pdf

Life sciences study design

All studies must disclose on these points even when the disclosure is negative.

Sample size	14
Data exclusions	We did not exclude any data
Replication	Repeated analyses produced the same results
Randomization	N/A
Blinding	N/A

Reporting for specific materials, systems and methods

We require information from authors about some types of materials, experimental systems and methods used in many studies. Here, indicate whether each material, system or method listed is relevant to your study. If you are not sure if a list item applies to your research, read the appropriate section before selecting a response.

Materials & experimental systems

n/a	Involved in the study
☒	☐ Antibodies
☒	☐ Eukaryotic cell lines
☐	☒ Palaeontology and archaeology
☒	☐ Animals and other organisms
☒	☐ Clinical data
☒	☐ Dual use research of concern

Methods

n/a	Involved in the study
☒	☐ ChIP-seq
☒	☐ Flow cytometry
☒	☐ MRI-based neuroimaging

Palaeontology and Archaeology

Specimen provenance

ROT: University of Tomsk
BOO: Anthropology department at Peter the Great Museum of Anthropology and Ethnography (Kunstkamera).

Specimen deposition

Indicate where the specimens have been deposited to permit free access by other researchers.

Dating methods

Radiocarbon dating (MAMS-57646)

☒ Tick this box to confirm that the raw and calibrated dates are available in the paper or in Supplementary Information.

Ethics oversight

Permissions to study the archaeological samples presented were obtained directly from collaboration partners and co-authors of this study: BOO individuals - permissions were acquired from Valery Khartanovich, head of the Anthropology department at Peter the Great Museum of Anthropology and Ethnography (Kunstkamera). ROT individuals - Permissions were acquired from Sergey Kuzminykh, Leading Researcher at the Laboratory of Natural Science Methods in Archaeology at the Russian Academy of Sciences and Igor Kovtun, University of Tomsk.

Note that full information on the approval of the study protocol must also be provided in the manuscript.
