# Supplementary material for: Bronze age Northern Eurasian genetics in the context of development of metallurgy and Siberian ancestry
Source: Commun Biol. 2024 Jun 11;7:723. doi: 10.1038/s42003-024-06343-x (PMC11166947; doi:10.1038/s42003-024-06343-x)
Supplement: Supplementary file 1 — Supplementary Information [file 42003_2024_6343_MOESM1_ESM.docx]

**Supporting Information for**

**Bronze Age Northern Eurasian Genetics in the Context of Development of Metallurgy and Siberian Ancestry**

Ainash Childebayeva, Fabian Fricke, Adam Benjamin Rohrlach, Lei Huang, Stephan Schiffels, Outi Vesakoski, Kristiina Mannermaa, Lena Semerau, Franziska Aron, Vyacheslav Moiseyev, Valery Khartanovich, Igor Kovtun, Johannes Krause, Sergey Kuzminykh, Wolfgang Haak

Ainash Childebayeva and Wolfgang Haak

Email: [ainash_childebayeva@eva.mpg.de](mailto:ainash_childebayeva@eva.mpg.de), [wolfgang_haak@eva.mpg.de](mailto:wolfgang_haak@eva.mpg.de)

**This PDF file includes:**

Supplementary Notes 1-4

Supplementary Figures 1-12

Supplementary Tables 1-6

Supplementary References

**Supplementary Note 1**

**Archaeological Background for the Seima-Turbino phenomenon.**

The name Seima-Turbino derives from the two eponymous burial grounds Seima and Turbino excavated in the beginning of the 20^th^ century CE^10^. The ST phenomenon combines elements of several cultures and does not represent a single culture in itself, especially since there are no distinct settlements or pottery styles associated with it. Instead, certain metal objects, which were found throughout Eurasia, from China and Central Asia in the East to Finland and Moldova in the West, spanning across approximately three million square kilometers, represent key ST-associated criteria (Fig. 1, and Supplementary Fig. 1). Briefly, the artifacts in the east contain higher amounts of tin (Sn) (Supplementary Fig. 2 and 3), and also more casting molds have been identified in the east compared to the west of the Urals, while a greater number and variation of ST objects have been found west of the Urals (Fig. 1c). The metal inventory of ST-complexes can be divided into two major groups: (1) objects that can be attributed to archaeological cultures of the Eurasian BA, such as Alakul, Abashevo, Sintashta, Petrovka and Srubnaya; and (2) objects that are only known from ST-sites (e.g., socket axes, lamellar dagger blades, fully hilted daggers and knives and the so-called forked lanceheads) (Fig. 1c). Singular finds of objects and weaponry of the ST type have been reported from contexts otherwise attributed to Bronze Age Glazkovo, Okunevo, Elunino, Odino, Krotovo, Koptyaki, Sintashta, Petrovka, Abashevo, Srubnaya (Pokrovka), and Post-Fatyanovo cultures^10,11^. However, these finds are rare and most ST materials are found at ST-associated sites.

ST burials are very distinct from those of the other North Eurasian cultures: individuals were buried mostly without pottery and not in kurgans, both inhumations and cremations were common, and the grave goods included bronze, stone, and bone weaponry, as well as bone armor. In cases where pottery is present at ST-sites, it can be attributed to other local cultures, for example Koptyaki at the site Shaitanskoe Ozero II^12^.

Generally, there is no drastic change in the metal complex, stone and bone inventory of the ST proper, however, some differences do exist. There are two traditions of manufacturing bronze celts: the Rostovka-Seima tradition (tools with and without hooks, decorated with a ladder belt and geometric figures - triangles and rhomboids), and the Turbino tradition (without side hooks, decorated with a belt of horizontal lines or without any ornamentation). Both traditions were continued, first of all, in the forest belt of Northern Eurasia in the post-Seima epoch until the end of the Early Iron Age.

Based on the analysis of ST artifacts (typology and metal alloy composition), a “western” (or European) and “eastern” (or Transural Siberian) variant of the phenomenon have been identified^10^. For example, in the east of the Urals a greater number of casting molds have been identified compared to the west of the Urals. However, west of the Urals a greater number and variation of ST objects have been identified. Of note, burials of actual metal workers (blacksmiths) have only been found east of the Urals. The western ST can also be characterized by the presence of the so-called Eurasian component potentially associated with Abashevo cultures and others from the Southern Urals and the Volga-Kama River basin (Chernykh and Kuzminykh 1989). Geographic structure regarding the degree of similarity of typology among the ST objects has been shown, with the similarity decreasing with distance^10^.

Regarding their regional attribution, the typical artifacts of the ST phenomenon can be categorized into three groups. First, there are artifact types that can be found in the entire distribution area of the ST phenomenon. Those are the lamellar dagger blades of the types NK-2, NK-4 and NK-6, the socket axes of the types K-14, K-18, K-30, K-32 and K-34 and the forked lanceheads of the types KD-12, KD-14, KD-16, KD-18, KD-20, KD-22, KD-24 and KD-26. The lamellar dagger blades have in common a flat cross section without a mid-rib. The different types are mainly based on the size of the blades themselves. The different types of socket axes are all relatively heavily decorated with rhombuses, triangles and tram-line ornaments around the socket. The different types of forked lanceheads are mainly defined by the presence or absence of ornaments and loops on the socket^10^.

Special to the area from the Urals to the western end of the ST distribution area are the lamellar dagger blades of the types NK-6 and NK-8, the serrated dagger blades of the types NK-18, NK-20, NK-22 and NK-24, the socket axes of the types K-4, K-6, K-8, K-10, K-12 and K-16 and the fully hilted daggers of the types KZh-2, KZh-4, KZh-6 and KZh-8. The lamellar dagger blades from NK-6 and NK-8 distinguish themselves by a rhombic cross section with a slide mid-rib on the blade. Most of the specific socket axes of the western part of the STP are less ornamented with the expectation of K-16, which was exclusively found in Seima on the banks of the Volga. The fully hilted daggers of the types KZh-2 and KZh-4 show lamellar ST-style blades. The daggers from the types KZh-6 and KZh-8 have curved blades, which are closer in style to dagger blades of the steppe cultures (Sintashta, Petrovka, Alakul etc.), but fully hilted the nearly exclusively appear in ST-contextes. Beside these ST-specific types, artifacts of the aforementioned Steppe-related cultures also appear more often in the western part of the distribution area^10^.

The eastern part of the ST phenomenon east of the foothills of the Ural Mountains also provides specific kinds of objects. The types K-20, K-22, K-24, K-26 and K-28 distinguish themselves mostly by a heavier ornamentation and a more trapezoidal shape. The forked lanceheads of the types KD-6, KD-8 and KD-10 are extraordinarily large compared to the other types of these lanceheads. Examples of K-10 moreover show a large hook on the side of the socket. The knife blades of the type NK-28 are special, as they were found mounted in antler handles at 60°-90° degrees. The blade-style of the fully hilted knives of the types KZh-8 and KZh-10 is unique within the ST phenomenon and is most likely the base of the later development of the knives of the Siberian Karasuk culture^10^.

To analyze the metallurgy of the aforementioned objects, we have to compare data that was obtained through very different methods during various periods of archaeometallurgic research. The oldest data stem from the very starting days of the Laboratory of Scientific Methods of the Academy of Science of the USSR from the early 1960s using Optically Stimulated Spectrometry. The newest data were obtained during the last decade in Russian and German laboratories operating on X-ray fluorescence. Because of that, we chose a rather robust approach, comparing just the most important alloying element for the metallurgy of the ST-era, tin (Sn).

We grouped all of the ST-sites in six different regional groups, following river systems and mountain ranges. The most western group is made up of the Baltic sites, however, there is no trace element analysis of this material. East of these sites, there is a concentration of ST-sites between the Dnipro and the Upper Volga. More to the east, there is a dense group of ST-related sites at the banks of the Middle and Lower Volga and the Kama. The agglomeration of ST-sites east of the Ural Mountains we describe as the Transural-Region. The area of the banks of the Ob and Irtysh rivers makes up another region, and finally the sites in the Altai and Tuva Mountains form the most eastward region (Supplementary Figure 1).

Going west to east, we observe a steady increase of Sn within ST-artifacts. The Dnipro/Upper Volga and Lower Volga/Kama regions are very comparable with a median of 2% Sn and 1% Sn and an upper end of the 1-sigma distribution around 6% Sn and lower end around 0% Sn each. The median of the Transural region is about 3% Sn and a slightly higher 1-sigma distribution of 1% to 7%. The objects of the Ob/Irtysh region though have a median of 8% Sn with a 1-sigma interval from 5% to 10% Sn. The Altai/Tuva region is even a bit higher with a median of 10% and a 1-sigma distribution from 7% to 11% Sn. The upper end of the 2-sigma interval for all regions is at about 15% Sn (Supplementary Figure 2).

The correlation between geographical location and the amount of Sn holds true when analyzed by typological groups (Supplementary Figure 3). For example, western-related socket ax types contain significantly less Sn compared to the eastern-related or nonspecific types. The difference between nonspecific and eastern-related types is smaller but is still recognisable. In the case of forked lanceheads, eastern-related types contain more Sn than nonspecific types (there are no specifically western-related types of forked lances). The ST-specific dagger blades are the exception to this rule. The few western-related dagger blades contain a higher amount of Sn than the unspecific ones (there are no eastern-related types of dagger blades), but they in general contain less Sn compared to forked lances or socket axes. These results once again confirm the theory that the Altai along with the other mountain ranges of Eastern Kazakhstan should be considered the source of Sn for the ST phenomenon.

Changes in the material complex of ST are connected with contacts and incorporations from other cultures. On the Irtysh River at the Rostovka site wrought spearheads and knives, typical for Sintashta and Petrovka cultures of the Trans-Urals steppes are found.

Turbino, Korshunovo, and Ust-Gaiva sites on the Middle Kama show the presence of axes, wrought spearheads, and knives. In Sokolovka at the mouth of the Kama River, in Ust-Vetluga at the mouth of the Vetluga River, in Seima and Reshnyi on the Oka River - axes, wrought spearheads, knives, and adornments typical for the Abashevo and Pokrovka (early Russian Srubnaya) antiquities are present.

Ceramics from the Siberian burial grounds of the ST (Rostovka and Satyga) show forms similar to the vessels of the Krotovo and other West Siberian cultures. In the Middle Kama burials and in Ust-Vetluga there are no ceramics in the burials. The vessels from Sokolovka, Seima and Reshnyi are Abashevo-like based on their shape (bell-shaped), ornamentation and alloy composition (shell).

Chernykh and Kuzminykh (1989) have proposed a hypothesis about the ST origins in the Altai-Xinjiang area in the Altai mountains and foothills, and in the cultures of the Baikal-Sayan area (e.g., Glazkovo). It is in these cultures that the initial examples of the bronze weaponry and ST-type objects are found. Both influences can be seen in the materials from Rostovka^10,13^, and it is possible that the people of the Baikal-Sayan area could also be the carriers of the proto-Uralic languages^10,13^.

**Supplementary Figure 1.** Regional groupings of the sites of the Seima-Turbino phenomenon.

**Supplementary Figure 2.** Tin-content of the artifacts of ST-sites grouped by regions (a) and cultural attribution (b).

**Supplementary Figure 3.** Tin-content of socket axes (a), forked lances (b) and lamellar dagger blades (c) grouped by cultural attribution of their types.

**Supplementary Note 2**

**Site descriptions**

Rostovka (ROT) is a ST burial site located on the river Om, in the city of Omsk, and was excavated in 1966-1969^1^. A total of 38 graves were excavated at the site, not all of which contained human remains. Some of the individuals buried at Rostovka were cremated, including ten clear cases of cremation, two graves with charcoal only, and one case with charcoal and the remains of children^2^.

In fact, the majority of Rostovka burials contain weapons, found in 60% of the female and 80% of the male burials^3^. The radiocarbon dates for Rostovka, excluding extreme values, range between ca. 2200-2000 cal. BCE^1,4,5^. This chronological horizon is simultaneous with the Okunevo culture, while its lower bound overlaps with the early Abashevo and Sintashta cultures, among others (Fig. 1b).

The burial ground of Rostovka occupies a very curious position within the ST phenomenon. It is the largest ST-site east of the foothills of the Ural Mountains. It is, compared to other important sites like Seima and Turbino, well excavated, documented and published. Rostovka is, together with the smaller sites of Kargat 6 and Preobrazhenka 6, the most eastern point of the distribution of steppe-related artifacts in ST-contexts. At these three sites also specifically, western ST-artifacts still appear, but in very small numbers. The ceramics of Rostovka are connected to the Krotovo and other forest-steppe cultures. The stone-artifacts are partly connected to the traditions of Baikalian stone-tools (little arrowheads, rectangular blades) partly to the western part of the ST phenomenon (bigger arrowheads, irregular blades) and in the case of a stone mace head to the Steppe-related cultures of the Transurals^2^. The metal composition of the artifacts of Rostovka is very comparable to the overall tendencies of the ST phenomenon. Steppe-related artifacts tend to contain less Sn than regional nonspecific ST-artifacts. And these nonspecific ST-artifacts contain less Sn than eastern ST-artifacts.

The burial site of Bolshoy Oleni Ostrov (BOO, also known as the Kola Oleneostrovskiy Cemetery KOC or the Kola Oleneostrovskiy Grave field, KOG) was first excavated in 1925, with the most recent excavation taking place between 2001-2004^6^. A total of N=43 individuals were found, along with wooden grave constructions, as well as bone, antler, stone, ceramic, and bronze items. Most burials were inhumations, with the exception of three cremations, and most individuals were buried in wooden boat-shaped caskets^6^.

Bolshoy Oleni Ostrov is situated in the Murmansk region, northwest part of the Russian Federation. The site is an island in the Kola Bay of the Barents Sea. Fifty-seven graves were observed at the cemetery, of which 25 were destroyed by the military in 1935. Archaeological excavations have uncovered 32 grave entities containing 43 individuals^6^. Most burials were inhumations, with the exception of three cremations, and most individuals were buried in wooden boat-shaped caskets^6^.

Excavations occurred in various periods: 1925 by G.D. Richter and S.F. Egorov (two graves), 1928 by A.V. Schmidt (eleven graves), 1947–1948 by N.N. Gurina (ten graves) and 1998–1999 and 2002–2004 by the Kola Archaeology Expedition IHMC RAS, led by V.Y. Shumkin (nine graves)^6,7^.

Radiocarbon dating indicates that the cemetery dates to the later Early Metal Period, specifically the second half of the 2nd millennium BC^6^. The BOO cemetery is very special due to unique preservation of organic materials, including human skeletal remains as well as animal osseous finds. A significant aspect of burial practices involved depositing artifacts in graves, such as various tools and weapons made of stone and bone, fish hooks, pins, combs, pendants and figurines made of bone and antler^7^. Ceramic shards, mainly of Lovozero and waffle (or textile) ornamented types were found in some graves^7^. Metal artifacts are rare in BOO graves, but a traceological and experimental study of a sample of bone objects from BOO by Anna Malyutina concluded that many of the artifacts were made using metal tools^8^.

A unique burial practice at BOO is that the deceased were buried in wooden caskets or containers, potentially boats or sledges. The type of sledge could be similar to those used by the historical Saami of Fennoscandia^6,7^. Unfortunately, due to a decayed state of the wood parts, it is impossible to determine what kind of containers they were. Most of the containers were covered with a wooden lid. Remains of birch bark and some other organic material was found above the skeletal remains, probably from animal furs or cloths covering the body.

The human bones from various excavations from the BOO cemetery are stored in the Peter the Great Museum of Anthropology and Ethnography MAE (Kunstkamera) of the Russian Academy of Sciences RAS (anthropological materials, museum numbers 4952, 5715, 7265) and the City Museum of Local History and Lore of the city of Polyarny (human remains from the grave 13, collection 5461/1 and 9002/I-V) (Kolpakov et al. 2019). MAE RAS also preserved archaeological materials (artifacts) collected from the BOO graves by Schmidt and Tsiplenkov (museum numbers 4082, 6152) and part of Nina Gurina´s excavations. Some artifacts from Gurina´s excavations belong to collections of the Murmansk Museum of Regional Studies (museum numbers 363, 364, 3133). The archaeological finds from the 2002–2004 excavations belong to collections of the City Museum of Local History and Lore of Polyarny (Murashkin et al. 2026; Malyutina 2019).

**Supplementary Note 3**

**Grave descriptions**

**Rostovka**

At the level of burials of the screened individuals from Rostovka there are also several observations, which should be recognized. Out of the nine graves with successfully analyzed individuals, five contain ST-style metal artifacts. The grave of ROT013 contained no finds at all^2^. The remains of individual ROT17 could not be attributed to any grave^2^.

Grave 5, in which ROT002 was buried, contained few finds. The inventory included a metal lance head (type KD-40), which is very typical for the site of Rostovka and is not widely distributed outside of the region, four little jasper fragments, and a stone arrow head^2^.

In Grave 7, where ROT003 was buried, only a few pieces of horn, bone and stone artifacts were found. Close the grave, a little asymmetrical quartz-blade and a piece of bone, which could be a fragment of a body-armor, like in grave 33, were found.^2^

Grave 8, in which ROT004 was buried, was particularly rich. It contained two lanceheads of the types KD-10 and KD-14, a socket ax of type K-20, a dagger blade of the type NK-6, several small flint arrowheads, two rectangular flint blades, and a bone handle^2^.

ROT006 was found in grave 10. It was a rather simple burial with a few asymmetrical stone blades and seven stone arrowheads. Close to the burial, a few ceramic fragments and a sharpening stone were found^2^.

ROT011 was buried in grave 24 together with a metal dagger blade (type NK-14), a metal awl, a fragment of a clay casting mould for an unidentified lancehead and a stone arrow head. Close to the burial, another concentration of finds was uncovered. Among them were: two lanceheads (one forked type KD-10, one KD-40), a socket ax (type K-32) and a little fragment of quartz^2^.

Burial 28 contained no finds aside from the remains of ROT013. In the area around it, only one ceramic fragment, a little metal awl and a small number of stone blades were found^2^.

The inventory of grave 33, in which ROT015 was buried, contained a lancehead of type KD-24, two gold rings, several small flint arrowheads, two stone objects, which are usually described as arrow-smoothers, and a big number of perforated bone battens. These battens are interpreted as the remains of a piece of body-armor^2^.

ROT016 was buried in grave 34 with an inventory of a dagger blade of type NK-4, a socket ax of type 20, a lancehead of type KD-10, two gold rings, a big ceramic fragment, several small flint blades, and a little bone awl^2^.

ROT017 was unfortunately not found in a clearly defined grave, but in a trench and no finds could be clearly associated with the individual^2^.

**Bolshoy Oleni Ostrov**

BOO001, Grave III. The single grave III grave was excavated in 1948 by A.V. Schmidt. Although the human skeleton is well-preserved, the grave was partially destroyed during excavation. The individual was aged as an adult and sexed to female by Yakimov (1953)^9^. Two bone knives were found—one on the pelvis and another on the stomach. Another bone object was discovered near the leg, along with a shell fragment and fragments of wood^7^. The human remains belong to the collection of the Peter the Great Museum of Anthropology and Ethnography MAE 5715:1.

BOO003, Grave 16, skeleton 1 and BOO004, grave 16, skeleton 4. The multiple grave 16 contains four burials, each placed in wooden constructions (possibly a boat or sledge) arranged in rows side by side. A rectangular cairn of boulders covered the grave. Most likely, burials were made separately in their own pits. The deceased include two females around 55–65 years, one male around 60, and one male around 45 years.

Skeleton 16-1, a female aged 55–65 years, was placed on her back in a wooden construction.The grave contained various finds, including wing bones of an unspecified auk (*Uria* sp./*Fratercula arctica*), sea scallop shell, a bone dagger and a bone handle with holes.

The skeleton 16-4, a 45-55 years-old male, was lying on his back inside a wooden construction. A cairn of stones covered the burial. The bottom of the construction was covered with wood-organic substance. Two double-pronged dart heads made of bone were found near the right knee. A set of artefacts, such as bone harpoon, antler adze, beaver (*Castor fiber*) tooth pendant, slate adze and a piece of grooved pumice were found on the right pelvic bones. A fragment of pottery was discovered in the fill above the right tibia^7^.

The grave 16 was excavated by the Kola Archaeology Expedition IHMC RAS, led by V.Y. Shumkin^7^. Human remains from these burials belong to the collection of the Peter the Great Museum of Anthropology and Ethnography MAE 7265:4 (16-1) and 7265:7 (16-4).

BOO005, Grave 17, skeleton 3 and BOO009, skeleton 4. The grave 17 contains burials of five individuals buried in separate wooden constructions (possibly a boat or sledge?). Burials were not made simultaneously, except 17-4 and 17-5. They were found in rows, in three separate grave pits dug next to each other. The deceased are one adult male (45–53 years; 17-1), a 10–12-year-old child (17-2), 35–45-year-old female (17-3), 16–18 years-old teenager (17-4) and a newborn baby (17-5). The baby 17-5 appears to have been placed in a small wooden container (possibly a cradle?) which, in turn, was placed inside the wooden container of the burial 17-4^7^.

The skeleton 17-3 belongs to a 35–45 years-old female and was found in the same grave pit as the child 17-2. It cannot be convincingly estimated whether the two individuals were buried at the same time, but they definitely share the same wooden construction. No artefacts or other finds were found by either skeleton.

The skeleton 17-4 belongs to a young female. The biological sex cannot be estimated osteologically, but our DNA analysis revealed that the deceased is a female. On the right side of her pelvis was a concentration of various finds: quartz flakes, antler and bone objects and a beaver incisor. On top of the cluster was an ornamented bone plate, most likely part of a bag mouthpiece. All the aforementioned items were most likely inside of this bag made of some decayed material. To the right of the skeleton 17-4 laid a skeleton of newborn baby (skeleton 17-5). On the pelvic area of the baby was a harpoon head. The grave was covered with a layer of decayed wood.

The grave 17 was excavated by the Kola Archaeology Expedition IHMC RAS, led by V.Y. Shumkin^7^. Human remains belong to the collection of the Peter the Great Museum of Anthropology and Ethnography MAE 7265:10 (17-3) and 7265:11 (17-4).

BOO006, Grave 19, skeleton 4. Grave 19 is a multiple grave of four adult individuals, one approximately one-year-old child, and one fetus. The skeletons were inside of wooden structures (possibly a boat or sledge) placed side by side in a row. The grave consists of three different pits made at different times. The child was in the same grave with a 55–60 years-old male (burial 19-2). Burial 19-3 of a woman of approximately 30 years of age had a skeleton of a fetus between the femora and pelvic bones.

The skeleton 19-4 belongs to a 55–60 years-old female. She was lying on her back inside a separate wooden construction, but in the same grave pit as 19-3. The burial contained a rich collection of grave goods, including antler rods with carved reindeer heads, slate, flint and quartz items, harpoons, fishhooks, shells, daggers, beaver and reindeer teeth, and a bone pendant. The grave appears to have been covered with a layer of birch bark.

The grave 19 was excavated in 2004 by the Kola Archaeology Expedition IHMC RAS, led by V.Y. Shumkin^7^. Human remains in 19-4 belong to the collection of the Peter the Great Museum of Anthropology and Ethnography MAE 7265:15.

BOO008, Grave 15, skeleton 1. Grave 15-1 comprises two wooden structures, possibly boat or sledge. One of these structures was empty, while the other contained a burial of a female aged 17–20 (the sex was confirmed in our DNA-analysis). Near the skull, a mandible of a wild reindeer (*Rangifer tarandus*), a broken sea scallop shell, and a piece of an antler tool were found. On the frontal bone, she had two bronze plaques. By the right arm, there was another wild reindeer jaw with teeth, a bone perforator, and a red ochre-coloured *Unio* shell. On pelvis, she had a flint dagger with birch bark, a bone dagger, and a bone comb. Between the two wooden structures a sea scallop shell was found.

The grave 15 was excavated in by the Kola Archaeology Expedition IHMC RAS, led by V.Y. Shumkin^7^. Human remains 15-1 belong to the collection of the Peter the Great Museum of Anthropology and Ethnography MAE 7265:3.

**Supplementary Figure 4. Cross-validation (CV) errors for each number of clusters (K) from the unsupervised ADMIXTURE analysis.**

**Supplementary Figure 5A. ROT002 outgroup *f*_3_-statistics with published modern and ancient populations.** Modern Uralic-speaking populations are shown as triangles, and modern non-Uralic speaking populations are shown as circles. Top five tests are indicated by population names. Only tests with more than 10,000 SNPs are shown.

**Supplementary Figure 5B. ROT003 outgroup *f*_3_-statistics with published modern and ancient populations.** Modern Uralic-speaking populations are shown as triangles, and modern non-Uralic speaking populations are shown as circles. Top five tests are indicated by population names. Only tests with more than 10,000 SNPs are shown.

**Supplementary Figure 5C. ROT004 outgroup *f*_3_-statistics with published modern and ancient populations.** Modern Uralic-speaking populations are shown as triangles, and modern non-Uralic speaking populations are shown as circles. Top five tests are indicated by population names. Only tests with more than 10,000 SNPs are shown.

**Supplementary Figure 5D. ROT006 outgroup *f*_3_-statistics with published modern and ancient populations.** Modern Uralic-speaking populations are shown as triangles, and modern non-Uralic speaking populations are shown as circles. Top five tests are indicated by population names. Only tests with more than 10,000 SNPs are shown.

Supplementary Figure 5E. ROT011 outgroup *f*_3_-statistics with published modern and ancient populations. Modern Uralic-speaking populations are shown as triangles, and modern non-Uralic speaking populations are shown as circles. Top five tests are indicated by population names. Only tests with more than 10,000 SNPs are shown.

Supplementary Figure 5F. ROT013 outgroup *f*_3_-statistics with published modern and ancient populations. Modern Uralic-speaking populations are shown as triangles, and modern non-Uralic speaking populations are shown as circles. Top five tests are indicated by population names. Only tests with more than 10,000 SNPs are shown.

**Supplementary Figure 5G. ROT015 outgroup f3-statistics with published modern and ancient populations.** Modern Uralic-speaking populations are shown as triangles, and modern non-Uralic speaking populations are shown as circles. Top five tests are indicated by population names. Only tests with more than 10,000 SNPs are shown.

**Supplementary Figure 5H. ROT016 outgroup *f*_3_-statistics with published modern and ancient populations.** Modern Uralic-speaking populations are shown as triangles, and modern non-Uralic speaking populations are shown as circles. Top five tests are indicated by population names. Only tests with more than 10,000 SNPs are shown.

**Supplementary Figure 5I. ROT017 outgroup *f*_3_-statistics with published modern and ancient populations.** Modern Uralic-speaking populations are shown as triangles, and modern non-Uralic speaking populations are shown as circles. Top five tests are indicated by population names. Only tests with more than 10,000 SNPs are shown.

**Supplementary Figure 5J. BOO001 outgroup *f*_3_-statistics with published modern and ancient populations.** Modern Uralic-speaking populations are shown as triangles, and modern non-Uralic speaking populations are shown as circles. Top five tests are indicated by population names. Only tests with more than 10,000 SNPs are shown.

**Supplementary Figure 5K. BOO02 outgroup *f*_3_-statistics with published modern and ancient populations.** Modern Uralic-speaking populations are shown as triangles, and modern non-Uralic speaking populations are shown as circles. Top five tests are indicated by population names. Only tests with more than 10,000 SNPs are shown.

**Supplementary Figure 5L. BOO03 outgroup *f*_3_-statistics with published modern and ancient populations.** Modern Uralic-speaking populations are shown as triangles, and modern non-Uralic speaking populations are shown as circles. Top five tests are indicated by population names. Only tests with more than 10,000 SNPs are shown.

**Supplementary Figure 5M. BOO04 outgroup *f*_3_-statistics with published modern and ancient populations.** Modern Uralic-speaking populations are shown as triangles, and modern non-Uralic speaking populations are shown as circles. Top five tests are indicated by population names. Only tests with more than 10,000 SNPs are shown.

**Supplementary Figure 5N. BOO05 outgroup *f*_3_-statistics with published modern and ancient populations.** Modern Uralic-speaking populations are shown as triangles, and modern non-Uralic speaking populations are shown as circles. Top five tests are indicated by population names. Only tests with more than 10,000 SNPs are shown.

**Supplementary Figure 5O. BOO06 outgroup *f*_3_-statistics with published modern and ancient populations.** Modern Uralic-speaking populations are shown as triangles, and modern non-Uralic speaking populations are shown as circles. Top five tests are indicated by population names. Only tests with more than 10,000 SNPs are shown.

**Supplementary Figure 5P. BOO008 outgroup *f*_3_-statistics with published modern and ancient populations.** Modern Uralic-speaking populations are shown as triangles, and modern non-Uralic speaking populations are shown as circles. Top five tests are indicated by population names. Only tests with more than 10,000 SNPs are shown.

**Supplementary Figure 5R. BOO009 outgroup *f*_3_-statistics with published modern and ancient populations.** Modern Uralic-speaking populations are shown as triangles, and modern non-Uralic speaking populations are shown as circles. Top five tests are indicated by population names. Only tests with more than 10,000 SNPs are shown.

**Supplementary Figure 6. Pairwise outgroup-*f*_3_ results.** **a** MDS plot of pairwise outgroup-*f*_3_ distances. Populations are indicated by shape and color. BOO individuals were analyzed separately. The rest of the *f*_3_ models were grouped by population label; **b** heatmap of pairwise outgroup-*f*_3_ distances; **c** heatmap of pairwise outgroup-*f*_3_ distances of BOO, ROT, and relevant modern populations. Modern populations are labeled using color according to their geographic location and language family.

**Supplementary Figure 7. Spatial covariance plot for the DATES admixture dating analysis for BOO when grouped together.** Admixture sources are: EEHG and Eastern Siberia LNBA.

**Supplementary Note 4**

**Demographic modeling of BOO with momi2**

4.1 Preface

Demographic modeling of BOO, admixed between Eastern European Hunter-Gatherer (EEHG) and Eastern Sibera LNBA, was performed using momi2^14^. The demographic model was inferred based on the joint site frequency spectrum (SFS), with the “momi2” algorithm^14^. The model is based on a phylogenetic tree, with population size changes within lineages and admixture events between lineages, which contain necessary demographic events. We used the “momi” package in Python^14^ to define models and fit parameters.

EEHG, which is represented by Sidelkino, is primarily of Western Eurasian ancestry, and Eastern Sibera LNBA, which is represented by Eastern_Siberia_LNBA, is primarily of Eastern Eurasian ancestry, but there is no clear-cut split between two ancestry sources due to the shared Ancient North Eurasian (ANE) ancestry. Therefore, in our model exploration, we involved Western and Eastern Eurasian lineages with no ANE ancestry, represented by Loschbour (WEHG) and CHB (Modern Han Chinese) respectively, to include sufficient background information.

All samples involved in the modeling are listed in Supplementary Table 13. Ancient samples were downloaded from the European Nucleotide Archive (ENA) as bam files, and then processed and imputed using GLIMPSE, following the default parameters^15^. Modern individuals were extracted from the 1000G database^16^. To generate SFS, we used all biallelic SNPs in 1000G.

**Supplementary Table 1. Sample information for modeling.**

| **Name in the model** | **Individual(s)** | **Population** | **Lineage** | **Age** | **Coverage** | **Ref.** |
| --- | --- | --- | --- | --- | --- | --- |
| Bolshoy | BOO004 | Early Metal Age population from Kola peninsula, Russia | Bolshoy Oleni Ostrov (BOO) | 3587 | 31.8x | this study |
| Sidelkino | Sidelkino | Eastern European Hunter-Gatherer | EEHG | 11250 | 2.9x | ^17^ |
| Eastern_  Siberia_  LNBA | yak022, yak024 | Late Neolithic/Bronze Age population from Kolyma River regions of Yakutia, Russia | Eastern Siberia LNBA | 3803 | 9.0x, 5.6x | ^18^ |
| YRI | NA18486, NA18488 | Yoruba in Ibadan, Nigeria | African | Modern | mean 8.2x for Africans in 1000G | ^16^ |
| Loschbour | Loschbour | Western European Hunter-Gatherer | Western Eurasian/WEHG | 8055 | 22x | ^19^ |
| CHB | NA18525, NA18526 | Han Chinese in Beijing, China | Eastern Eurasian / East Asian | Modern | mean 7.7x for East Asians in 1000G | ^16^ |

In order to fit the model parameters quickly and accurately, we progressively added populations into the model. The exploration of the model structure is a tree search. For each step, there could be multiple model structure definitions. For each model structure definition, we initialized the parameters before fitting, copying old parameters from previous models and randomly generating new parameters within reasonable upper and lower bounds. We initialized the new parameters multiple times (100 for the final model, 20 for others), and ran the optimization algorithm respectively to search for the parameters with maximum log-likelihoods, using that as the basis for the next step. The final decision of the model was based on the log-likelihood of the model, the model complexity and the rationality based on other lines of evidence.

There were 3 steps in the modeling procedure. We denoted the model in each step as follows:

Step 1: We fitted the model involving Western Eurasian lineage (Loschbour) and Eastern Eurasian lineage (CHB) as a simple split, with EEHG (Sidelkino) added onto the Western Eurasian lineage (Loschbour), Eastern Siberia LNBA (Eastern_Siberia_LNBA) added onto Eastern Eurasian lineage (CHB), and modern African individuals (YRI) used as an outgroup.

Step 2: Based on the models with highest likelihoods from Step 1, we represented ANE ancestry existing in both EEHG (Sidelkino) and Eastern Sibera LNBA (Eastern_Siberia_LNBA).

Step 3: Based on modeling from Step 2, we fitted the BOO lineage (Bolshoy) as an admixture of EEHG (Sidelkino) and Eastern Sibera LNBA (Eastern_Siberia_LNBA).

We only considered autosomal mutations and assumed a mutation rate of 1.25×10^-8^ per site per generation^20^ and a generation time of 29 years^21^. We set the diploid effective size of 1.2×10^4 14^, and the autosomal genome length to 2,881,033,286 base pairs.

4.2 Step 1

The initial model involved 5 populations with the phylogeny (YRI, ((Loschbour, Sidelkino), (Eastern_Siberia_LNBA, Han))). Different models (1-4) had different definitions on the population size.

Model 1: We defined different population sizes for all internal lineages.

Model 2: We defined the same population size for the ancestral lineage of (Loschbour, Sidelkino), (Eastern_Siberia_LNBA, Han) and ((Loschbour, Sidelkino), (Eastern_Siberia_LNBA, Han)).

Model 3: We defined the same population size for Loschbour and the ancestral lineage of (Loschbour, Sidelkino), and the same population size for Han and the ancestral lineage of (Eastern_Siberia_LNBA, Han).

Model 4: From the split of (Loschbour, Han) to Loschbour and Han liniages respectively, we defined exponential population size change starting from ancestral Eurasian population size.

We selected the models with highest log-likelihood (Supplementary Fig. 8; Supplementary Table 14). Model 1 and 4 were chosen for the next step.

                            Model 1                                                Model 2

                            Model 3                                                Model 4

**Supplementary Figure 8. Momi2 models with highest log-likelihood (Model 1-4).** Point estimates of the final model are shown in blue. The sampling times of populations are indicated by circles. The population sizes are indicated by the thickness of branches. The y-axis is linear below 10,000 years ago, and logarithmic above it. See Supplementary Table 14 for specific parameter values.

**Supplementary Table 2. Parameter estimates of the models with highest log-likelihoods (Models 1-4).** N: effective population size; t: time; p: admixture proportion; (A, B): the ancestor of A and B (B branches off from A); A→B: the admixture pulse from A to B.

| **Parameter** | **Model 1** | **Model 2** | **Model 3** | **Model 4** |
| --- | --- | --- | --- | --- |
| log-likelihood | -38980945 | -38980980 | -38982025 | -38980974 |
| N_YRI_ | 5.42e+04 | 5.43e+04 | 5.41e+04 | 5.41e+04 |
| N_Loschbour_ | 1.73e+03 | 1.45e+03 | 2.15e+03 | 1.44e+03 |
| N_CHB_ | 1.48e+04 | 1.53e+04 | 6.73e+03 | 1.78e+04 |
| t_(YRI, Loschbour)_ | 8.98e+04 | 9.00e+04 | 8.84e+04 | 8.87e+04 |
| t_(Loschbour, CHB)_ | 4.76e+04 | 4.75e+04 | 5.38e+04 | 4.98e+04 |
| N_(Loschbour, CHB)_ | 3.05e+03 | 3.07e+03 | 2.47e+03 | 2.80e+03 |
| N_(Loschbour, Sidelkino)_ | 2.26e+03 | =N_(Loschbour, CHB)_ | =N_Loschbour_ | - |
| N_(CHB, Eastern_Siberia_LNBA)_ | 3.26e+03 | =N_(Loschbour, CHB)_ | =N_CHB_ | - |
| N_(YRI, Loschbour)_ | 1.75e+04 | 1.75e+04 | 1.75e+04 | 1.75e+04 |
| t_(Loschbour, Sidelkino)_ | 3.51e+04 | 3.06e+04 | 4.17e+04 | 3.59e+04 |
| N_Sidelkino_ | 4.76e+03 | 3.87e+03 | 6.07e+03 | 4.91e+03 |
| t_(CHB, Eastern_Siberia_LNBA)_ | 2.72e+04 | 2.82e+04 | 1.32e+04 | 2.15e+04 |
| N _Eastern_Siberia_LNBA_ | 2.48e+03 | 2.59e+03 | 1.01e+03 | 1.90e+03 |

4.3 Step 2

In this step, we modeled the ANE ancestry shared between the Sidelkino and Eastern_Siberia_LNBA lineages. For simplicity, we did not use specific samples such as MA1^22^ to model the ANE ancestry. The shared ANE ancestry in Sidelkino and Eastern_Siberia_LNBA was modeled as gene flow between Western and Eastern Eurasians.

Models 1.1 and 4.1: We defined gene flow from CHB to Sidelkino, as well as gene flow from Sidelkino to Eastern_Siberia_LNBA.

Models 1.2 and 4.2: We defined gene flow from CHB to Sidelkino, as well as gene flow from Loschbour to Eastern_Siberia_LNBA.

Models 1.3 and 4.3: We defined gene flow from Eastern_Siberia_LNBA to Sidelkino, as well as gene flow from Sidelkino to Eastern_Siberia_LNBA.

Models 1.4 and 4.4: We defined gene flow from Eastern_Siberia_LNBA to Sidelkino, as well as gene flow from Loschbour to Eastern_Siberia_LNBA.

If the Eastern Eurasian gene flow was from CHB, it could be earlier or later than the divergence of Eastern_Siberia_LNBA. Similarly, if the Western Eurasian gene flow was from Loschbour, it could be earlier or later than the divergence of Sidelkino. We also didn’t add constraints on the order of gene flows between Western and Eastern Eurasians. The optimization algorithm was used to automatically decide the best model for each model definition.

We tried four different versions of gene flow on the basis of Model 1 (Supplementary Figure 9; Supplementary Table 15) and Model 4 (Supplementary Figure 10; Supplementary Table 16). Models 1.2 and 4.2 had the lowest log-likelihood among the four models. Also, all models based on the substrate of Model 1 were better than those of the corresponding Model 4. We chose Models 1.1, 1.3, 1.4 and 4.1 for the next step.

                            Model 1.1                                               Model 1.2

                            Model 1.3                                               Model 1.4

**Supplementary Figure 9. Momi2 models with highest log-likelihoods (Models 1.1-1.4).** Point estimates of the final model are shown in blue. The sampling times of populations are indicated by circles. The population sizes are indicated by the thickness of branches. The y-axis is linear below 10,000 years ago, and logarithmic above it. See Supplementary 15 for specific parameter values.

**Supplementary Table 3. Parameter estimates of the models with highest log-likelihoods (Models 1.1-1.4).** N: effective population size; t: time; p: admixture proportion; (A, B): the ancestor of A and B (B branches off from A); A→B: the admixture pulse from A to B.

| **Parameter** | **Model 1.1** | **Model 1.2** | **Model 1.3** | **Model 1.4** |
| --- | --- | --- | --- | --- |
| log-likelihood | -38977134 | -38977631 | -38977011 | -38977130 |
| N_YRI_ | 5.44e+04 | 5.45e+04 | 5.44e+04 | 5.44e+04 |
| N_Loschbour_ | 1.66e+03 | 1.42e+03 | 1.65e+03 | 1.31e+03 |
| N_CHB_ | 3.76e+04 | 5.41e+04 | 3.39e+04 | 3.81e+04 |
| t_(YRI, Loschbour)_ | 8.95e+04 | 9.09e+04 | 8.97e+04 | 9.01e+04 |
| t_(Loschbour, CHB)_ | 5.29e+04 | 4.93e+04 | 5.20e+04 | 5.12e+04 |
| N_(Loschbour, CHB)_ | 2.77e+03 | 3.17e+03 | 2.85e+03 | 2.95e+03 |
| N_(Loschbour, Sidelkino)_ | 2.96e+03 | 2.99e+03 | 2.90e+03 | 3.58e+03 |
| N_(CHB, Eastern_Siberia_LNBA)_ | 3.57e+03 | 2.44e+03 | 3.33e+03 | 2.95e+03 |
| N_(YRI, Loschbour)_ | 1.75e+04 | 1.75e+04 | 1.75e+04 | 1.75e+04 |
| t_(Loschbour, Sidelkino)_ | 3.26e+04 | 2.91e+04 | 3.27e+04 | 2.77e+04 |
| N_Sidelkino_ | 3.61e+03 | 3.18e+03 | 3.42e+03 | 2.52e+03 |
| t_(CHB, Eastern_Siberia_LNBA)_ | 2.42e+04 | 2.91e+04 | 2.56e+04 | 2.77e+04 |
| N _Eastern_Siberia_LNBA_ | 1.93e+03 | 2.42e+03 | 2.12e+03 | 2.31e+03 |
| p_CHB/ Eastern_Siberia_LNBA →Sidelkino_ | 0.0917 | 0.0802 | 0.0727 | 0.0711 |
| t_CHB/ Eastern_Siberia_LNBA →Sidelkino_ | 3.03e+04 | 2.91e+04 | 1.62e+04 | 1.13e+04 |
| p_Loschbour/Sidelkino→ Eastern_Siberia_LNBA_ | 0.129 | 0.134 | 0.107 | 0.110 |
| t_Loschbour/Sidelkino→ Eastern_Siberia_LNBA_ | 2.24e+04 | 2.91e+04 | 2.56e+04 | 2.77e+04 |

                            Model 4.1                                                        Model 4.2

                            Model 4.3                                                        Model 4.4

**Supplementary Figure 10. Momi2 models with highest log-likelihoods (Models 4.1-4.4).** Point estimates of the final model are shown in blue. The sampling times of populations are indicated by circles. The population sizes are indicated by the thickness of branches. The y-axis is linear below 10,000 years ago, and logarithmic above it. See Supplementary Table 16 for specific parameter values.

**Supplementary Table 4. Parameter estimates of the models with highest log-likelihoods (Models 4.1-4.4).** N: effective population size; t: time; p: admixture proportion; (A, B): the ancestor of A and B (B branches off from A); A→B: the admixture pulse from A to B.

| **Parameter** | **Model 4.1** | **Model 4.2** | **Model 4.3** | **Model 4.4** |
| --- | --- | --- | --- | --- |
| log-likelihood | -38977412 | -38978884 | -38977362 | -38977611 |
| N_YRI_ | 5.41e+04 | 5.41e+04 | 5.41e+04 | 5.41e+04 |
| N_Loschbour_ | 1.80e+03 | 1.71e+03 | 1.78e+03 | 1.81e+03 |
| N_CHB_ | 2.24e+04 | 1.99e+04 | 2.23e+04 | 2.20e+04 |
| t_(YRI, Loschbour)_ | 8.82e+04 | 8.83e+04 | 8.82e+04 | 8.81e+04 |
| t_(Loschbour, CHB)_ | 5.43e+04 | 5.35e+04 | 5.39e+04 | 5.44e+04 |
| N_(Loschbour, CHB)_ | 2.56e+03 | 2.62e+03 | 2.57e+03 | 2.57e+03 |
| N_(YRI, Loschbour)_ | 1.75e+04 | 1.75e+04 | 1.75e+04 | 1.75e+04 |
| t_(Loschbour, Sidelkino)_ | 3.73e+04 | 3.51e+04 | 3.81e+04 | 3.76e+04 |
| N_Sidelkino_ | 4.39e+03 | 3.89e+03 | 4.21e+03 | 3.51e+03 |
| t_(CHB, Eastern_Siberia_LNBA)_ | 1.60e+04 | 1.96e+04 | 1.73e+04 | 2.00e+04 |
| N _Eastern_Siberia_LNBA_ | 1.22e+03 | 1.62e+03 | 1.36e+03 | 1.65e+03 |
| p_CHB/ Eastern_Siberia_LNBA →Sidelkino_ | 0.114 | 0.174 | 0.0640 | 0.109 |
| t_CHB/ Eastern_Siberia_LNBA →Sidelkino_ | 3.70e+04 | 3.51e+04 | 1.14e+04 | 1.13e+04 |
| p_Loschbour/Sidelkino→ Eastern_Siberia_LNBA_ | 0.101 | 0.0482 | 0.0789 | 0.0477 |
| t_Loschbour/Sidelkino→ Eastern_Siberia_LNBA_ | 1.60e+04 | 1.96e+04 | 1.73e+04 | 2.00e+04 |

4.4 Step 3

At last, we added the highest coverage BOO individual BOO004 (Bolshoy) into the models from the previous step, as an admixture of Sidelkino and Eastern_Siberia_LNBA. We defined the ghost lineage for two source populations, with the same population size as their original branch, and modeled the Bolshoy lineage with its own population size as the admixture of ghost lineages.

For Models 1.3 and 1.4, the model failed to converge after adding the Bolshoy lineage. The optimization of the basis of Model 1.1 and 4.1 was successful (denoted as Model 1.1.1 and 4.1.1). For both Model 1.1.1 and 4.1.1, we got a series of similar likelihood results with recent admixture time and small population size in Bolshoy (see Supplementary Tables 10-12) and chose the final model with the admixture time matching the conclusion from DATES (yellow background in Supplementary Table 10; Supplementary Figure 11; Supplementary Table 17). The ancestry proportions and the effective population size of the Bolshoy lineage were similar for both models, but Model 1.1.1 had a higher log-likelihood, which we chose as our final model.

To examine the stability of the parameters, we conducted 100 nonparametric bootstraps for Model 1.1.1, fit the model for each bootstrap dataset using the parameter values of the final model as initial values, and computed the 95% confidence interval for each parameter (Supplementary Figure 12; Supplementary Table 18). Each bootstrap dataset was created by dividing the whole genome into 100 equal-sized blocks and resampling the same number of blocks with replacement. It turned out that the median was close to the parameter estimates.

                        Model 1.1.1                                          Model 4.1.1

**Supplementary Figure 11. Momi2 models with highest log-likelihoods (Models 1.1.1 and 4.1.1).** Point estimates of the final model are shown in blue. The sampling times of populations are indicated by circles. The population sizes are indicated by the thickness of branches. The y-axis is linear below 10,000 years ago, and logarithmic above it. See Supplementary Table 17 for specific parameter values.

**Supplementary Table 5. Parameter estimates of the models with highest log-likelihoods (Models 1.1.1 and 4.1.1**). N: effective population size; t: time; p: admixture proportion; (A, B): the ancestor of A and B (B branches off from A); A→B: the admixture pulse from A to B.

| **Parameter** | **Model 1.1.1** | **Model 4.1.1** |
| --- | --- | --- |
| log-likelihood | -44302812 | -44303272 |
| N_YRI_ | 5.35e+04 | 5.32e+04 |
| N_Loschbour_ | 1.18e+03 | 1.60e+03 |
| N_CHB_ | 3.18e+04 | 1.85e+04 |
| t_(YRI, Loschbour)_ | 8.779e+04 | 8.700e+04 |
| t_(Loschbour, CHB)_ | 5.301e+04 | 5.202e+04 |
| N_(Loschbour, CHB)_ | 2.66e+03 | 2.66e+03 |
| N_(Loschbour, Sidelkino)_ | 3.99e+03 | 1.75e+04 |
| N_(CHB, Eastern_Siberia_LNBA)_ | 3.91e+03 | 3.433e+04 |
| N_(YRI, Loschbour)_ | 1.75e+04 | - |
| t_(Loschbour, Sidelkino)_ | 2.518e+04 | - |
| N_Sidelkino_ | 2.47e+03 | 3.93e+03 |
| t_(CHB, Eastern_Siberia_LNBA)_ | 2.158e+04 | 1.480e+04 |
| N _Eastern_Siberia_LNBA_ | 1.69e+03 | 1.11e+03 |
| p_CHB→Sidelkino_ | 0.0936 | 0.129 |
| t_CHB→Sidelkino_ | 2.518e+04 | 3.431e+04 |
| p_Sidelkino→ Eastern_Siberia_LNBA_ | 0.125 | 0.0942 |
| t_Sidelkino→ Eastern_Siberia_LNBA_ | 1.923e+04 | 1.480e+04 |
| N_Bolshoy_ | 235 | 190 |
| t_Ghost_Siberia→Bolshoy_ | 4137 | 4114 |
| p_Ghost_Siberia→Bolshoy_ | 0.398 | 0.398 |
| t_(Eastern_Siberia_LNBA, Ghost_Siberia)_ | 1.148e+04 | 8573 |
| t_(Sidelkino, Ghost_Sidelkino)_ | 1.902e+04 | 2.360e+04 |

**Supplementary Figure 12. Momi2 model with the highest log-likelihood (Model 1.1.1) with bootstrapping.** Point estimates of the final model are shown in blue; results for 100 nonparametric bootstraps are shown in gray. The sampling times of populations are indicated by circles. The population sizes are indicated by the thickness of branches. The y-axis is linear below 10,000 years ago, and logarithmic above it. See Supplementary Table 18 for specific parameter values.

**Supplementary Table 6. Parameter estimates of Model 1.1.1, along with the statistics of parameters in 100 nonparametric bootstrap estimations (SD: standard deviation; percentage: quantile).** N: effective population size; t: time; p: admixture proportion; (A, B): the ancestor of A and B (B branches off from A); A→B: the admixture pulse from A to B.

| **Parameter** | **Estimate** | **50%** | **SD** | **2.5%** | **97.5%** |
| --- | --- | --- | --- | --- | --- |
| N_YRI_ | 5.35e+04 | 5.40e+04 | 2.87e+03 | 4.82e+04 | 5.83e+04 |
| N_Loschbour_ | 1.18e+03 | 1.23e+03 | 175 | 935 | 1.65e+03 |
| N_CHB_ | 3.18e+04 | 3.50e+04 | 1.76e+04 | 1.88e+04 | 8.44e+04 |
| t_(YRI, Loschbour)_ | 8.779e+04 | 8.807e+04 | 1.60e+03 | 8.525e+04 | 9.104e+04 |
| t_(Loschbour, CHB)_ | 5.301e+04 | 5.262e+04 | 1.68e+03 | 4.920e+04 | 5.554e+04 |
| N_(Loschbour, CHB)_ | 2.66e+03 | 2.70e+03 | 215 | 2.31e+03 | 3.13e+03 |
| N_(Loschbour, Sidelkino)_ | 3.99e+03 | 3.86e+03 | 513 | 2.78e+03 | 4.70e+03 |
| N_(CHB, Eastern_Siberia_LNBA)_ | 3.91e+03 | 3.90e+03 | 368 | 3.15e+03 | 4.54e+03 |
| N_(YRI, Loschbour)_ | 1.75e+04 | 1.74e+04 | 126 | 1.72e+04 | 1.77e+04 |
| t_(Loschbour, Sidelkino)_ | 2.518e+04 | 2.582e+04 | 2.21e+03 | 2.224e+04 | 3.044e+04 |
| N_Sidelkino_ | 2.47e+03 | 2.75e+03 | 516 | 1.93e+03 | 3.79e+03 |
| t_(CHB, Eastern_Siberia_LNBA)_ | 2.158e+04 | 2.129e+04 | 1.60e+03 | 1.860e+04 | 2.481e+04 |
| N _Eastern_Siberia_LNBA_ | 1.69e+03 | 1.66e+03 | 180 | 1.38e+03 | 2.02e+03 |
| p_CHB→Sidelkino_ | 0.0936 | 0.0951 | 0.0262 | 0.0436 | 0.147 |
| t_CHB→Sidelkino_ | 2.518e+04 | 2.582e+04 | 2.21e+03 | 2.224e+04 | 3.044e+04 |
| p_Sidelkino→ Eastern_Siberia_LNBA_ | 0.125 | 0.132 | 0.0195 | 0.0777 | 0.157 |
| t_Sidelkino→ Eastern_Siberia_LNBA_ | 1.923e+04 | 1.921e+04 | 2.09e+03 | 1.458e+04 | 2.273e+04 |
| N_Bolshoy_ | 235 | 206 | 92.7 | 118 | 441 |
| t_Ghost_Siberia →Bolshoy_ | 4137 | 4089 | 145 | 3778 | 4357 |
| p_Ghost_Siberia →Bolshoy_ | 0.398 | 0.397 | 0.0254 | 0.349 | 0.444 |
| t_(Eastern_Siberia_LNBA, Ghost_Siberia)_ | 1.148e+04 | 1.137e+04 | 1.03e+03 | 9310 | 1.296e+04 |
| t_(Sidelkino, Ghost_Sidelkino)_ | 1.902e+04 | 1.904e+04 | 1.56e+03 | 1.652e+04 | 2.205e+04 |

**Supplementary References**

1.	Marchenko, Z. V., Svyatko, S. V., Molodin, V. I., Grishin, A. E. & Rykun, M. P. Radiocarbon Chronology of Complexes With Seima-Turbino Type Objects (Bronze Age) in Southwestern Siberia. *Radiocarbon* **59**, 1381–1397 (2017).

2.	Matyushenko, V. I. & Sinitsina, G. V. Могильник у д. Ростовка вблизи Омска [Burial Ground near the Village of Rostovka near Omsk]. https://elibrary.ru/item.asp?id=24232508 (1988).

3.	Chernykh, E. N. *et al.* Issues in the calendar chronology of the seima-turbino transcultural phenomenon. *Archaeol. Ethnol. Anthropol. Eurasia (Russ.-lang.)* **45**, 45–55 (2017).

4.	Ковтун, И. В., Марочкин, А. Г. & Герман, П. В. Радиоуглеродные даты и относительная хронология сейминско-турбинских, крохалёвских и самусьских древностей. *Труды V (XXI) Всероссийского* (2017).

5.	Hanks, B. K., Epimakhov, A. V. & Renfrew, A. C. Towards a refined chronology for the Bronze Age of the southern Urals, Russia. *Antiquity* **81**, 353–367 (2007).

6.	Murashkin, Kolpakov & Shumkin. Kola Oleneostrovskiy grave field: a unique burial site in the European Arctic. *Iskos* (2016).

7.	Kolpakov, E. M., Murashkin, A. I. & Khartanovich, V. I. Kola Oleneostrovsky cemetery (1925-2013). https://elibrary.ru/item.asp?id=41653832 (2019).

8.	Malyutina, A. Experiment and traceology. *In* 436–459 (2019).

9.	Якимов, В. П. Антропологическая характеристика костяков из погребений на Большом Оленьем острове (Баренцево море). *Сб. МАЭ* (1953).

10.	Chernykh, E. N. & Kuzminykh, S. V. Древняя металлургия Северной Евразии (сейминско-турбинский феномен). https://elibrary.ru/item.asp?id=21143678 (1989).

11.	Кузьминых, С. В. Сейминско-турбинская проблема: новые материалы. *Краткие сообщения Института археологии* 240–263 (2011).

12.	Korochkova, O. *Sacred Place of the First Metallurgists in the Middle Ural*. (Издательство Уральского университета, 2020).

13.	Кузьминых, С. В. Сейминско-турбинский транскультурный феномен: формирование, развитие и исторические судьбы. *и миграции: концепции, методы, результаты/Отв …* (2019).

14.	Kamm, J., Terhorst, J., Durbin, R. & Song, Y. S. Efficiently inferring the demographic history of many populations with allele count data. *J. Am. Stat. Assoc.* **115**, 1472–1487 (2020).

15.	Rubinacci, S., Ribeiro, D. M., Hofmeister, R. J. & Delaneau, O. Efficient phasing and imputation of low-coverage sequencing data using large reference panels. *Nat. Genet.* **53**, 120–126 (2021).

16.	1000 Genomes Project Consortium *et al.* A global reference for human genetic variation. *Nature* **526**, 68–74 (2015).

17.	Damgaard, P. de B. *et al.* 137 ancient human genomes from across the Eurasian steppes. *Nature* **557**, 369–374 (2018).

18.	Kılınç, G. M. *et al.* Human population dynamics and Yersinia pestis in ancient northeast Asia. *Sci Adv* **7**, (2021).

19.	Lazaridis, I. *et al.* Ancient human genomes suggest three ancestral populations for present-day Europeans. *Nature* **513**, 409–413 (2014).

20.	Fu, Q. *et al.* Genome sequence of a 45,000-year-old modern human from western Siberia. *Nature* **514**, 445–449 (2014).

21.	Fenner, J. N. Cross-cultural estimation of the human generation interval for use in genetics-based population divergence studies. *Am. J. Phys. Anthropol.* **128**, 415–423 (2005).

22.	Raghavan, M. *et al.* Upper Palaeolithic Siberian genome reveals dual ancestry of Native Americans. *Nature* **505**, 87–91 (2014).

**Data used for ST plotting distribution of ST artifacts (Supp. Figures 1-3):**

1.	Degtyareva, A. D., and S. V. Kuzminykh. 2011. “Результаты аналитического исследования металлических изделий могильника Сатыга XVI [Results of Analytical Study of Metal Products of Satyga Burial Site XVI].” In *Сатыга XVI: Cейминско-турбинский могильник в таежной зоне Западной Сибири*, 37–44.

2.	Kuzminykh, S. V., V. Y. Lunkov, and L. B. Orlovskaya. 2017. “Результаты рентгенофлуоресцентного анализа: серия 2013-2016 гг [Results of X-Ray Fluorescence Analysis: Series 2013-2016].” In *Аналитические исследования лаборатории естественнонаучных методов.*, 34–60. elibrary.ru.

3.	Lunkov, V. Y., S. V. Kuzminykh, and L. B. Orlovskaya. 2011. “Рентгено-флуоресцентный анализ меди и бронз: серия 2009--2010 гг [X-Ray Fluorescence Analysis of Copper and Bronzes: Series 2009-2010].” In *Аналитические исследования лаборатории естественнонаучных методов*, 116–36. elibrary.ru.

4.	Lunkov, V. Y., S. V. Kuzminykh, and L. B. Orlovskaya. 2013. “Результаты ренгтгено-флуоресцентного анализа: Серия 2011-2013 гг [Results of X-Ray Fluorescence Analysis: Series 2011-2013].” *Аналитические исследования лаборатории естественнонаучных методов*, 56–88.

5.	Lunkov, V. Y., L. B. Orlovskaya, and S. V. Kuzminykh. 2009. “Рентгено-флуоресцентный анализ: начало исследований химического состава древнего металла [X-Ray Fluorescence Analysis: The Beginning of Research into the Chemical Composition of Ancient Metal].” In *Аналитические исследования лаборатории естественнонаучных методов*, 84–110. elibrary.ru.
